# Vibrational Spectra and Molecular Vibrational Behaviors of Dibenzyl Disulfide, Dibenzyl Sulphide and Bibenzyl

**DOI:** 10.3390/ijms23041958

**Published:** 2022-02-10

**Authors:** Ziyi Wang, Ruimin Song, Weigen Chen, Jianxin Wang, Pinyi Wang, Zhixian Zhang, Xinyuan Zhang, Fu Wan

**Affiliations:** State Key Laboratory of Power Transmission Equipment & System Security and New Technology, Chongqing University, Chongqing 400044, China; wzycsust@163.com (Z.W.); ruiminsong@cqu.edu.cn (R.S.); wang.jianxin@cqu.edu.cn (J.W.); wang.pinyi@outlook.com (P.W.); zhang.zhixian@cqu.edu.cn (Z.Z.); 18846116694@163.com (X.Z.); fuwan@cqu.edu.cn (F.W.)

**Keywords:** Raman spectroscopy, infrared spectroscopy, corrosive sulfur, density functional theory, vibration assignment

## Abstract

The vibration spectroscopy (Raman and infrared) of widely concerned molecules in sulfur corrosion phenomenon (Dibenzyl Disulfide, Dibenzyl Sulphide, and Bibenzyl) is detailedly analyzed based on density functional theory and experimental measurement. The dominant conformations of these molecules are determined according to Boltzmann distribution in relative Gibbs free energy. Additionally, noncovalent interaction analysis is conducted to indicate intramolecular interaction. Vibration normal mode is assigned based on potential energy distribution, which comprehensively reveals the molecular vibrational behaviors. Conformations weighted spectra are obtained and compared with experimentally measured spectra. We found that experimental spectra are in good agreement with the theoretical spectra in B3LYP-D3(BJ)/6-311G** level with a frequency correction factor. Furthermore, the divergence among these molecules is discussed. The vibrational behavior of the methylene group in the molecule shows a trend with the presence of the sulfur atom.

## 1. Introduction

**Figure 1:**
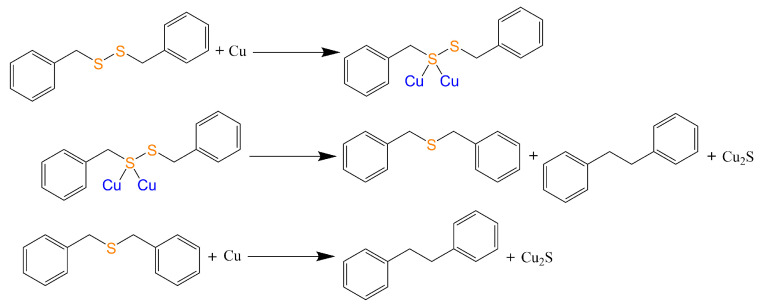
The corrosion mechanism of DBDS in power equipment.

Nowadays, the sulfur corrosion problem of power equipment has received widespread concern [1,2,3,4,5]. The failures caused by corrosive sulfur greatly affect the safety and stability of the power system and result in substantial economic losses. Dibenzyl Disulfide (DBDS) is regarded as the most important corrosive sulfur. The corrosion mechanism was proposed by Mitsubishi Electric Corporation as shown in Figure 1 [6]. The intermediate product Dibenzyl Sulphide (DBS) is also corrosive, and the final product of this reaction is Bibenzyl (BiBz) and cuprous sulfide. The DBDS, DBS, and BiBz are considered as indicators in power equipment sulfur corrosion phenomenon. The study on their physical and chemical properties will contribute to the development of advanced corrosive resistant materials and the realization of corrosive state monitoring.

Numerous efforts have been made to study properties of the DBDS, DBS, and BiBz, including chemical reactivity [7,8], synergistic effect [9,10], detection methods [6,11], etc. [12,13]. In this paper, the vibrational spectra and molecular vibrational behaviors are analyzed detailedly. The vibrational spectroscopy includes infrared and Raman spectroscopy, which is based on periodic changes of dipolmoments (IR) or polarizabilities (Raman) caused by molecular vibrations behaviors. IR and Raman spectra are both widely used methods for composition analysis and trace detection [14,15,16].

In addition to experimentally measured vibrational spectra, density functional theory (DFT) provides a solid foundation of theoretically calculated spectra. Considering the presence of a large number of fundamentals and overtones bands, it is difficult to interpret experimental spectra of large molecule [17]. However, the conformational distribution and theoretical spectrum of molecules can be obtained based on DFT. Then, a potential energy distribution (PED) based analysis can be performed to achieve vibration assignment, which gives a clear and crispy concept about the structural–vibrational properties of the molecule. A lot of studies have recently appeared in the literature concerning the calculation of spectroscopic properties by DFT methods [17,18,19,20,21,22].

## 2. Experimental Section

A commercially available Fourier transform infrared spectroscopy (FTIR, Nicolet iS50 FT-IR) was utilized for experimental test. The DBDS (CAS:150-60-7, 98%, Aladdin, China), DBS (CAS:538-74-9, 99%, Aladdin, China), and BiBz (CAS:103-29-7, 99%, Aladdin, China) samples were mixed with KBr (CAS:7758-02-3, 99.5%, Aladdin, China) and ground in a agate mortar for 1 min. Then, the mixed sample was pressurized by a tablet press at 20 MPa for 2 min for transmission testing. The IR spectra were measured between 400 cm${}^{-1}$ to 4000 cm${}^{-1}$ at a 4 cm${}^{-1}$ resolution. The spectra were an average of 32 scans.

A confocal Raman spectrometer mentioned in our previous paper [23] was used to detect experimental spectra of the DBDS, DBS, and BiBz. There are three kinds of gratings (600 lines/mm, 1200 lines/mm, and 1800 lines/mm) built in the spectrometer. The grating with minor grooves number is designed for wide spectral range detection, although the spectral resolution will decrease. In order to compare with theoretical spectra in full spectral range, 600 lines/mm grating was adopted. The other test parameters of the measurement are listed below: the slit width was 50 um, the integration time was 1 s, and the integration number was 20 times. The Raman platform was calibrated with monocrystalline silicon before each test. The Raman shift of the first-order silicon peak was adjusted to 520.7 cm${}^{-1}$. The result of Raman spectrum comes from the average of three measurements.

The Raman/IR spectra of DBDS, DBS, and BiBz were plotted in Section 4 after normalization and de-baseline operations.

## 3. Molecular Structure and Intramolecular Interaction

### 3.1. Conformation Determination

Flexible molecules always have multiple conformations, such as DBDS, DBS, and BiBz studied in this paper. The proportion of different conformations was calculated according to Boltzmann distribution in relative Gibbs free energy. The molecular conformation search program Molclus [24] is used for conformation determination in this paper.

Firstly, a batch of initial conformations of DBDS, DBS, and BiBz were generated by rotating chemical bond via Molclus. The chemical bond within the benzene ring was ignored; only C-C bonds, C-S bonds, and S-S bonds between benzene rings are considered. All the considered bonds rotate every 120°. The DBDS with five rotation bonds generates a total of $3^{5}$ initial conformations. Similarly, DBS generates $3^{4}$ initial conformations. BiBz generates $3^{3}$ initial conformations.

All initial conformations generated above were pre-optimized geometrically by Gaussian [25] in a semi-empirical method PM7 [26]. The optimized conformations with the energy difference within 0.25 kcal/mol and the geometric difference within 0.1 Å will be classified as the same conformation. The conformations with relatively lower energy were applied further geometric optimization and frequency analysis in B3LYP-D3(BJ)/6-311G** [27,28,29,30,31] level. For an authentic conformation distribution, a double hybrid functional revDSD-PBEP86-D3(BJ) [32] in conjunction with may-cc-PVTZ [33] was introduced to recalculate the single point energy. The single point can be added with thermal correction to find the precise Gibbs free energy. The thermal correction is obtained in frequency analysis based on rigid-rotor harmonic oscillator (RRHO) model [34] under 298.15K and 1 atm. The results are shown in Table 1.

**Figure 2:**
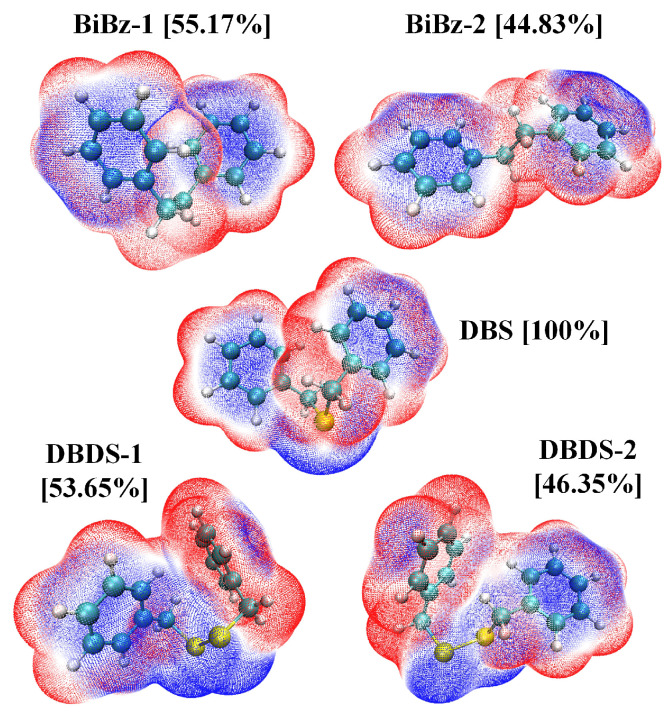
The geometric structure and surface electrostatic potential of the dominant conformation.

Conformations in Table 1 with a proportion less than 5% were ignored. The geometric structures and surface electrostatic potential of several dominant conformations were visualized via VMD [35], as shown in Figure 2. The electrostatic potential involved was evaluated by Multiwfn, based on the highly effective algorithm proposed in ref. [36].

It can be found from the surface electrostatic potential distribution that the negative charge region mainly occurs around the sulfur atom and the benzene ring planar center. It is difficult to explain the geometric structure only by the surface electrostatic potential distribution due to the existence of various intramolecular interaction. Further NCI analysis is thus needed.

### 3.2. Noncovalent Interaction Analysis of the Dominant Conformation

To reveal the nature of these dominant conformations with low free energy, the noncovalent interaction (NCI) [37] method was applied for weak molecular interactions analysis.

The NCI method uses Reduced Density Gradient (RDG) to distinguish regions with noncovalent interactions in the system. The expression of RDG is as follows, where ρ is the electron density.

(1) $$\mathrm{RDG}=\frac{1}{2\cdot \sqrt[{3}]{3\pi^{2}}}\cdot \frac{\left|\nabla \rho \right|}{\rho^{4/3}}$$

**Figure 3:**
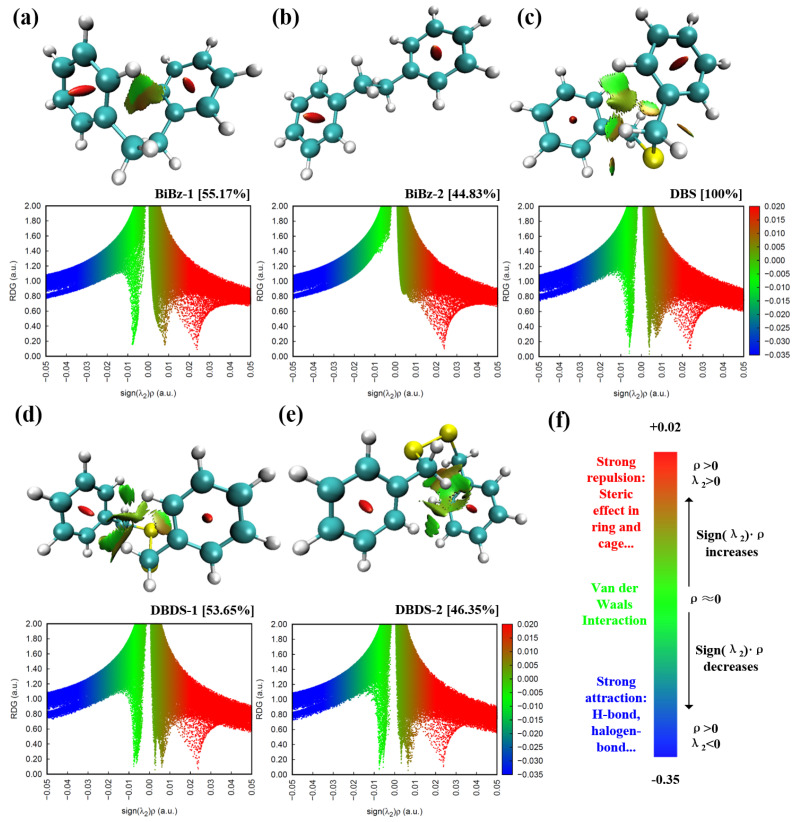
NCI analysis of dominant conformation. (**a**) RDG isosurface map and scatter plot of BiBz-1, (**b**) Bibz-2, (**c**) DBS, (**d**) DBDS-1, and (**e**) DBDS-2. (**f**) color map and chemical explanation.

The noncovalent interactions areas can be determined by RDG value. However, the strength and type of interaction are determined by ρ and $sign(\lambda_{2})$, respectively. The $sign(\lambda_{2})$ is the second largest eigenvalue of the electron density Hessian matrix. Overlapping the color map of $sign(\lambda_{2})\cdot \rho$, as shown in Figure 3, on the RDG value isosurface, means the areas, intensity, and type of the noncovalent interactions can be visualized at the same time.

The isosurface map and scatter plot of five dominant conformations were visualized via Multiwfn [38,39] and VMD [35]. As described in Figure 3f, the red area in the isosurface map and the scatter plot indicates the repulsion between molecules, such as steric effect. The green area indicates the van der Waals interaction, while the blue area indicates hydrogen bonds and halogen bonds that have a significant attraction.

There are two common features in the five dominant conformations. Firstly, significant steric effect is observed in the two benzene rings. This steric effect is shown as a red spindle-shaped area in the benzene ring on the RDG isosurface map, and as a red spike on the right side in the scatter plot. Secondly, there is no notable attraction in these conformations.

For BiBz-1, the van der Waals interaction is formed between the two benzene rings. The molecular structure of BiBz-2 is stretched, the two benzene rings are far apart, and no significant van der Waals effect is observed. This was shown as two spikes near the zero point on the scatter plot. The spikes can be positive or negative as the sign of $sign(\lambda_{2})\cdot \rho$ is relatively unstable as the electron density in the van der Waals area is small. The BiBz-1 formed by two benzene rings attracting each other is more stable and has a lower single point energy due to intermolecular forces. However, as BiBz-2 has a lower thermal correction, the Gibbs free energy and proportion of these two conformations are not remarkably different.

For DBS, in addition to the zone between benzene rings, the van der Waals effect also occurs between the sulfur atom and the hydrogen atom on the benzene ring. However, this kind of H-S interaction is distinguished from hydrogen bonds (shown in blue). The RDG isosurface between the two benzene rings in DBDS is much more complicated, but in conclusion, it is still van der Waals interaction. These broken isosurfaces were shown as many fine spikes near the zero point on the scatter plot in Figure 3d,e.

## 4. Vibration Analysis and Vibrational Spectra

### 4.1. PED Based Vibrational Assignment

Each characteristic group in molecules has its own unique vibration behavior. However, these groups will be coupled with the molecular systems, which change frequency and intensity. Non-linear molecules have 3n−6 normal vibration modes, where n is the number of atoms in the molecule. The normal modes are the coupling of multiple groups vibration. It is difficult to directly identify which groups and vibration types contribute to the normal mode.

PED (potential energy distribution) analysis is an effective method that decomposes the normal vibration mode into group characteristic vibrations. The contribution proportion of the group vibration can be obtained, and the vibrational features of normal mode can be revealed. In this paper, PED based vibrational assignment of DBDS, DBS, and BiBz was performed via VEDA4 program [40].

**Figure 4:**
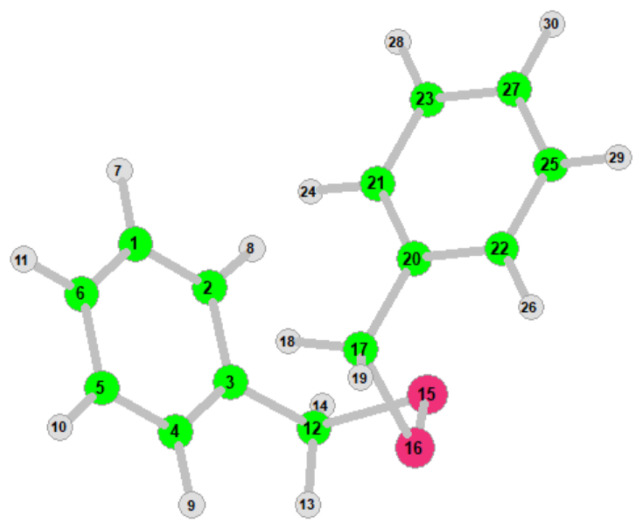
The atomic number of DBDS in VEDA4.

The non-linear molecule DBDS consists of 30 atoms, it has a total of 84 normal modes. Therefore, 84 linearly independent internal coordinates are introduced to describe molecular vibration. The internal coordinates and related atoms of the DBDS-1 conformation are shown in Table 2. Atom numbers are listed in Figure 4. Additionally, the internal coordinates and related information of other four conformations can be found in Supplementary Material. The vibration type in VEDA4 program are divided into stretching (ν), bending (δ), and torsion (τ/γ), which correspond to specific internal coordinates. The stretching vibration involves two atoms. Bending vibration involves three atoms and torsion vibration involves four atoms. Among them, torsion can be further divided into two types: τ and γ. In many cases, the two definitions can describe the same atoms movement, but not always. τ ABCD means the dihedral angle between the ABC and BCD planes. γ ABCD means the angle between the AD vector and the BCD plane (reffed from VEDA4 user document).

The internal coordinates of DBDS-1 conformation consist of 29 kinds of stretching, 28 kinds of bending, and 27 kinds of torsion. The contribution of different internal coordinates to the normal vibration mode is shown in Table 3 and Table 4. Similarly, the detailed table of other conformations is provided in Supplementary Material. It should be noted that the internal coordinates contribution less than 10% are ignored. The normal modes of DBDS-1 are divided into low frequency (<400 cm${}^{-1}$), middle frequency (400∼2800 cm${}^{-1}$), and high frequency (>2800 cm${}^{-1}$). The scaled frequency was corrected by a factor of 0.9640 [41].

Modes #1∼#14 are high frequency normal modes. All of them are contributed by stretching vibration, and there is a large frequency gap between #14 and #15. They have completely different characteristics in the frequency band. This is because the force constant of the stretching vibration is larger. The stretching vibration energy is greater than bending and torsion vibration energy. Therefore, the characteristic frequencies corresponding to the stretching vibration are all located in the high frequency region. Generally, almost all molecules have abundant vibration peaks corresponding to stretching vibrations in the high frequency region. The main feature of the low frequency zone is that no stretching contribution is assigned, while the middle frequency area is composed of various vibration contributions. The spectrum characteristics in the low frequency region is ignored in the following analysis. As the measurement range of FTIR only covers 400–4000 cm${}^{-1}$, and the range of Raman spectroscopy in the low frequency zone is seriously affected by Rayleigh scattering.

The normal modes always appear in pairs. This is because DBDS is a quasi-symmetric molecule. Although the DBDS-1 conformation does not have molecular symmetry, its molecular composition is still symmetrical. For example, the vibration mode of one benzene ring must have the corresponding vibration mode of another benzene ring.

#### 4.1.1. Methylene(-CH_2_) Group

In the high frequency zone, the asymmetric stretching vibration of the two -CH2 groups corresponds to the #11 and #12 normal modes. The two mode peaks appear at 3007 cm${}^{-1}$ and 3025 cm${}^{-1}$ respectively, due to the vibration coupling (the calculated Raman and IR spectra are shown in Figures S4 and S5, respectively). The infrared activity of #12 mode is weak, almost invisible, in the IR spectrum. The symmetric stretching vibration of the two -CH2 groups corresponds to the #13 and #14 normal modes. These two modes appear as the same peak at 2946 cm${}^{-1}$. The frequency of -CH2 symmetrical vibration is often smaller than that of asymmetrical vibration [21].

In the middle frequency zone, the scissoring vibration of -CH2 appears at 1416 cm${}^{-1}$, corresponding to mode #23 and #24. When wagging vibration of -CH2 appears at 1223 cm${}^{-1}$, corresponding to mode #29 and #30. High Raman activity and infrared activity are simultaneously exhibited in wagging vibration. The twisting vibration of -CH2 corresponds to #37 and #38 at 1126 cm${}^{-1}$. The rocking vibration of -CH2 corresponds to #51 and #52 at 863 cm${}^{-1}$. The activity of these four modes is relatively weak.

For the -CH2 group, the order of normal vibration frequency from large to small is: asymmetric stretch, symmetric stretch, scissoring, wagging, twisting, and rocking.

#### 4.1.2. Phenyl(-C_5_H_6_) Group

In the high frequency zone, the #1 and #2 normal modes correspond to the in-phase stretching vibration of the C-H bond on the benzene ring. For both #1 and #2 modes, the five C-H bonds on the benzene ring will vibrate in the synchronous phase. Two modes appear as the same peak at 3077 cm${}^{-1}$. This characteristic peak is the strongest peak in the Raman spectrum, but not in the infrared spectrum. The eight #3∼#10 normal modes correspond to the asynchronous phase vibration of the C-H bonds on the benzene ring, and the Raman activity and infrared activity of these eight modes are quite different. Modes #3 and #4 appear at 3069 cm${}^{-1}$ in the IR spectrum. Modes #5 and #6 appear at 3062 cm${}^{-1}$ in both the IR and Raman spectrum. Modes #7 and #8 appear as a peak at 3051 cm${}^{-1}$ in the Raman spectrum.

In the middle frequency zone, the stretching vibration of the carbon skeleton of the benzene ring corresponds to modes #15∼#18, #27, and #28. The frequency of these stretching vibration modes is much smaller than the C-H bond stretching vibration. This shows that the vibration force constant of the carbon skeleton is much smaller than the force constant of the C-H bond. The bending vibration of the hydrogen atoms on the benzene ring corresponds to #19∼#22, #25, #26, #33∼#36, #39, and #40. Modes #31 and #32 have very strong coupling characteristics. All atoms except disulfide bonds are involved, and both apparent carbon skeleton stretching and hydrogen atoms bending occurred.

The #41 mode (frequency < 1016 cm${}^{-1}$) began to exhibit bending of the benzene ring carbon skeleton The bending and stretching vibrations of the carbon skeleton of the benzene ring simultaneously appear in the #41 and #42 modes. The #43, #44, #55, #56, and #63∼#66 modes correspond to bending vibration of benzene ring carbon skeleton. High Raman activity is exhibited in these two modes, appearing as peak at 986 cm${}^{-1}$, which is also the characteristic peak used for Raman spectroscopy based DBDS detection in our study in progress.

From the #45 mode (frequency < 967 cm${}^{-1}$), the out-of-plane bending of hydrogen atoms on benzene ring occurs. Modes #45∼#50, #53 and #54, and #57∼#60 correspond to this type of vibration. Modes #67∼#69 correspond to the deformation vibration of the benzene ring couple with the stretching vibration of disulfide bond. The other out-of-plane vibration of the benzene ring appeared in the low frequency zone.

The #61 and #62 modes are composed of multiple vibrations, mainly including the in-plane bending, out-of-plane bending of the benzene ring, and the stretching vibration of C-S bond.

### 4.2. Weighted Vibrational Spectra

Different conformations have their own vibrational spectra. The weighed spectrum was calculated by different weighted conformations spectra according to their proportion. Frequency calculation is carried out in the optimal conformations; there is no imaginary frequency observed in the result. A frequency correction factor (0.9640) for B3LYP/6-311G** [41] was applied to the vibration spectra of DBDS, DBS, and BiBz due to the overestimated frequency of anharmonic effect.

**Figure 5:**
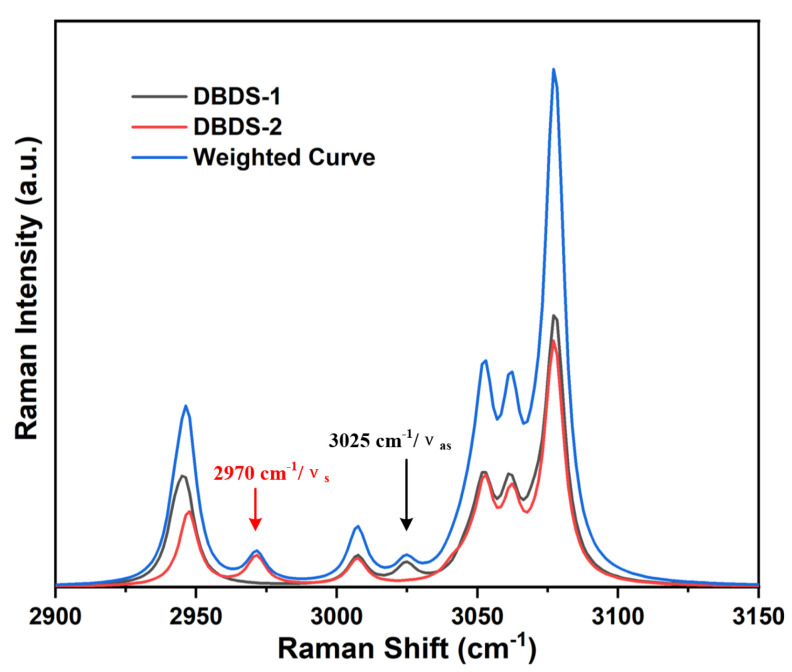
Theoretically calculated conformations Raman spectra and weighted curve of DBDS.

Take the Raman spectra of DBDS as an example to illustrate the role of weighted spectrum. Figure 5 shows the Raman spectra of two DBDS conformations in the high frequency zone. The most significant difference between the two conformations is that DBDS-1 has a characteristic peak at 3025 cm${}^{-1}$, but DBDS-2 has a characteristic peak at 2970 cm${}^{-1}$.

Based on the vibration assignment in Section 4.1, the peak at 3025 cm${}^{-1}$ is assigned to asymmetrical stretching vibration of two -CH_2_ groups in DBDS-1. The peak at 2970 cm${}^{-1}$ is assigned to symmetric stretching of -CH_2_ group in DBDS-2. The Raman spectra of different conformations are different, but they are all superimposed in the weighted curve, as shown in Figure 5’s blue line. The vibration spectroscopy is revealed much better when based on conformation weighted curve.

It should be noted that the Raman spectrum obtained by theoretical calculation is a spectrum of Raman activity (unit in Å${}^{4}$/amu). In order to compare with the experimental spectrum, the Raman activity is converted into the Raman intensity, depending on the frequency of incident light and ambient temperature [42]:

(2) $$I_{i}=\frac{C{\left(v_{o}-v_{i}\right)}^{4}S_{i}}{v_{i}B_{i}}$$

(3) $$B_{i}=1-exp\left(-\frac{hcv_{i}}{kT}\right)$$

where $S_{i}$, $I_{i}$, and $v_{i}$ are the Raman activity, Raman intensity, and vibration frequency of *i*th vibration mode respectively. $v_{0}$ is the frequency of incident light. *T* is the ambient temperature, *h* is Planck’s constant, *c* is the speed of light, *k* is Boltzmann’s constant, and *C* is an arbitrary constant. The conversion process above was conducted via Multiwfn code [38]. The laser source wavelength used in our experimental platform is 532 nm, and room temperature was set as 298.15 K.

### 4.3. Comparison of Theoretical and Experimental Spectra

The experimentally measured and theoretically calculated spectra of DBDS, DBS, and BiBz are provided in Supplementary Material. It can be found from the Figures S4–S9 that the experimental Raman spectrum is in good agreement with the theoretical spectrum. Generally, the B3LYP hybrid functional combining with 6-311G** basis set overestimates the vibrational frequency in the high vibrational frequency zone but underestimates the frequency in the low frequency zone. This phenomenon is consistent with previous studies [43]. Dual scaling factor in the high frequency zone and low frequency zone is optional for agreement improvement.

**Figure 6:**
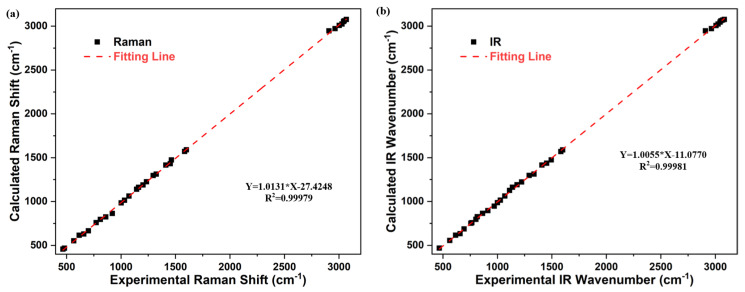
Linear fitting between theoretical and experimental Raman shift.

In order to quantitatively describe the consistency between the theoretical spectrum and the experimental spectrum, a linear fitting operation is performed to each characteristic peak of DBDS. The abscissa of peak data is the experimental frequency, and the ordinate is the theoretical frequency. The peak data fitting line close to y=x indicates a better agreement between the experimental value and the theoretical value. As shown in Figure 6, the fitting lines of Raman spectrum and infrared spectrum both show good linearity. The goodness of fit reaches 0.99979 and 0.99981, respectively. A good agreement is revealed at B3LYP-D3(BJ)/6-311G** level as the slope of the fitted line is close to 1.

The mode identification is shown in Table 5 and Table 6. Most experimental Raman peaks can be identified to corresponding normal modes within a reasonable deviation. There are also some normal vibration modes which are not measured in the experimental spectrum due to the weak Raman activity or infrared activity.

Different conformations contribute to different experimental peaks. For example, in the both Raman and infrared experimental spectra, the peak at 2964 cm${}^{-1}$ is only generated by the #13 normal mode of DBDS-2, while the #13 normal mode of DBDS-1 contribute to the peak at 2908 cm${}^{-1}$ together with the #14 mode. The characteristic peaks generated by a single conformation are marked with parentheses in Table 5 and Table 6.

In addition to the fundamental frequency band in the vibrational spectroscopy, there are many anharmonic phenomenon such as overtone, combination band, Fermi resonance, etc. Which contribute to several characteristic peaks in experiment spectrum that are not observed in the theoretical spectrum. For example, two peaks are observed in experimental Raman spectrum at 1495 cm${}^{-1}$ and 1526 cm${}^{-1}$. While the adjacent peaks at 1463 cm${}^{-1}$ and 1584 cm${}^{-1}$ correspond to the #19, #20, #17, and #18 mode, respectively. There is no corresponding theoretical normal mode for these two peaks (1495 and 1526) assignment. They are actually a combination band of peaks at 1002 cm${}^{-1}$ and 481 cm${}^{-1}$. The peak at 1002 cm${}^{-1}$ (#43 + #44) corresponds to in-plane bending vibration of benzene ring carbon skeleton, while the peak at 481 cm${}^{-1}$ (#67 + #68) is corresponding to the out-of-plane deformation vibration of the benzene ring. This kind of highly correlated vibration is prone to coupling and generating combined frequency band. Fermi resonance most often occurs between fundamental and overtone (combination) excitations, if they are nearly coincident in energy. The two peaks (1495 and 1526) are thus split under Fermi resonance with the action of strong fundamental frequency band (1600 cm${}^{-1}$).

### 4.4. Comparison of DBDS, DBS, and BiBz

**Figure 7:**
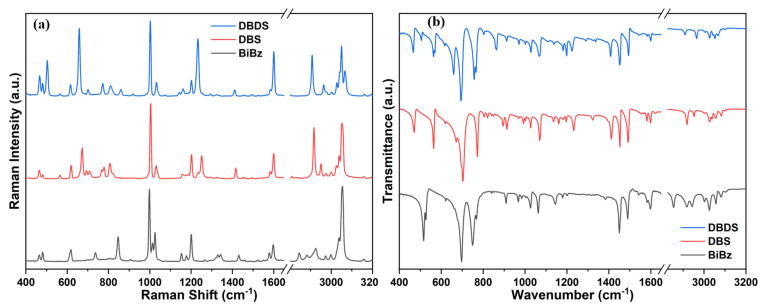
Experimental measured Raman (**a**) and IR (**b**) spectra of DBDS, DBS, and BiBz.

DBDS, DBS, and Bibz have remarkable similarities in molecular structure. The only difference is the number of sulfur atoms between the two benzyl groups. The electronegativity of sulfur atoms is stronger than that of carbon atoms and hydrogen atoms. The sulfur atom will cause the redistribution of electron as discussed in Section 3.1, which changes the molecular vibration frequency. The spectra comparison of DBDS, DBS, and BiBz is shown in Figure 7. In order to reveal the similarity and difference of the vibrational behaviors of the three molecules, the six vibrational behaviors of the methylene groups is analyzed.

The frequency distributions of these six vibrations are generally consistent. The order from largest to smallest is asymmetric stretching, symmetric stretching, scissoring, wagging, twisting, and rocking, according to the frequency magnitude.

**Figure 8:**
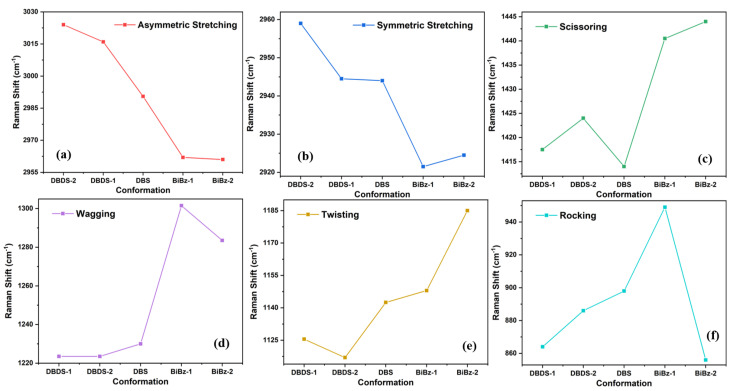
Tendency of different vibration behavior frequencies in the dominant conformations of DBDS, DBS, and BiBz. (**a**) Asymmetric Stretching; (**b**) Symmetric Stretching; (**c**) Scissoring; (**d**) Wagging; (**e**) Twisting; (**f**) Rocking.

The characteristic vibration frequency of six different vibration behaviors in the DBDS, DBS, and BiBz is shown in Table 7. The trend is plotted in Figure 8 and the frequency is averaged by two dual vibrations. It can be found that, for the stretching vibration located in the high frequency region, when the sulfur atom in the middle of the benzyl group is lost, the stretching vibration frequency tends to decrease, while the four bending vibration frequencies in the middle frequency region tend to increase.

The most interesting aspect of these graphs is the low rocking vibration value of BiBz-2, which shows a different trend from several other conformations. This is because the frequency difference between the two dual vibration is very large. BiBz-2 is the only molecule with $C_{2h}$ symmetry among the five dominant conformations. The rocking vibration at 748 cm${}^{-1}$ and 964 cm${}^{-1}$ are synchronous rocking and asynchronous rocking, respectively. The synchronous rocking at 748 cm${}^{-1}$ keeps symmetry during the vibration based on the center plane of two benzene rings. The polariseability change rate of this vibration is zero, thus the Raman activity is zero. Exhibiting extremely low frequency.

## 5. Conclusions

In this paper, the vibration spectroscopy (Raman and infrared) and molecular vibrational behaviors of widely concerned molecules in sulfur corrosion phenomenon (Dibenzyl Disulfide, Dibenzyl Sulphide, and Bibenzyl) is detailedly analyzed. First, the proportion of different conformations are calculated according to Boltzmann distribution in relative Gibbs free energy via Molclus program. In order to obtain an accurate Gibbs free energy, the single-point energy is calculated in revDSD-PBEP86-D3(BJ)/may-cc-PVTZ and thermodynamic correction is calculated in the B3LYP-D3(BJ)/6-311G** level, respectively. Five dominant conformations of these three molecules were obtained, and their intramolecular interactions were detailedly discussed by NCI analysis.

PED based vibrational assignment of DBDS, DBS, and BiBz was performed via VEDA4 program, the normal vibration modes are assigned. Conformations weighted spectra is compared with experimentally measured spectra. It can be found that experimental spectra are in good agreement with the theoretical spectra in the B3LYP-D3(BJ)/6-311G** level with a frequency correction factor (0.9640). The characteristic peak in experimental spectra is also identified to corresponding modes within a reasonable deviation. The overtone due to the anharmonic effect is also pointed out. The divergence among these molecules is discussed. It can be found that, for the stretching vibration of methylene group located in the high frequency region, when the sulfur atom in the middle of the benzyl group lost, the stretching vibration frequency tends to decrease, while the four bending vibration frequencies in the middle frequency region tend to increase.

In summary, the results presented in this paper provide a theoretical and experimental guidance for understanding the vibrational spectra and molecular vibrational behaviors of these molecules. We also believe that this article will lay a solid foundation for the development of advanced corrosive resistant materials and realization of the corrosive state monitoring.

## Figures and Tables

**Table 1:** Energy and conformation proportion of DBDS, DBS, and BiBz.

Molecule	Conformation	Single Point Energy (a.u.)	Thermal Correction (a.u.)	Gibbs Free Energy (a.u.)	Proportion
BiBz	1	−541.04114860	0.198497	−540.8426516	55.17%
	2	−541.03965980	0.197204	−540.8424558	44.83%
DBS	1	−938.9390301	0.196498	−938.7425321	91.45%
	2	−938.9372458	0.197657	−938.7395888	4.50%
	3	−938.9338298	0.194142	−938.7396878	4.05%
DBDS	1	−1336.847129	0.195781	−1336.651348	53.12%
	2	−1336.847175	0.195965	−1336.65121	45.89%
	3	−1336.844026	0.197072	−1336.646954	0.51%
	4	−1336.843633	0.196717	−1336.646916	0.49%

**Table 2:** Internal coordinates and related atoms in DBDS-1.

Stretching	Related Atoms	Bending	Related Atoms	Torsion	Related Atoms
ν1	C(1)-H(7)	δ30	C(22)-C(25)-C(27)	τ58	H(7)-C(1)-C(2)-C(3)
ν2	C(2)-H(8)	δ31	C(3)-C(12)-S(15)	τ59	H(8)-C(2)-C(1)-C(6)
ν3	C(4)-H(9)	δ32	H(7)-C(1)-C(2)	τ60	H(9)-C(4)-C(5)-C(6)
ν4	C(5)-H(10)	δ33	H(8)-C(2)-C(1)	τ61	H(10)-C(5)-C(6)-C(1)
ν5	C(6)-H(11)	δ34	H(9)-C(4)-C(5)	τ62	H(11)-C(6)-C(1)-C(2)
ν6	C(12)-H(13)	δ35	H(10)-C(5)-C(6)	τ63	H(13)-C(12)-S(15)-S(16)
ν7	C(12)-H(14)	δ36	H(11)-C(6)-C(1)	τ64	H(14)-C(12)-S(15)-S(16)
ν8	C(17)-H(18)	δ37	H(13)-C(12)-S(15)	τ65	H(18)-C(17)-S(16)-S(15)
ν9	C(17)-H(19)	δ38	H(14)-C(12)-H(13)	τ66	H(19)-C(17)-S(16)-S(15)
ν10	C(21)-H(24)	δ39	H(18)-C(17)-S(16)	τ67	H(24)-C(21)-C(23)-C(27)
ν11	C(22)-H(26)	δ40	H(19)-C(17)-H(18)	τ68	H(26)-C(22)-C(25)-C(27)
ν12	C(23)-H(28)	δ41	H(24)-C(21)-C(23)	τ69	H(28)-C(23)-C(27)-C(25)
ν13	C(25)-H(29)	δ42	H(26)-C(22)-C(25)	τ70	H(29)-C(25)-C(27)-C(23)
ν14	C(27)-H(30)	δ43	H(28)-C(23)-C(27)	τ71	H(30)-C(27)-C(25)-C(22)
ν15	C(2)-C(1)	δ44	H(29)-C(25)-C(27)	τ72	C(2)-C(1)-C(6)-C(5)
ν16	C(22)-C(25)	δ45	H(30)-C(27)-C(25)	τ73	C(22)-C(25)-C(27)-C(23)
ν17	C(1)-C(6)	δ46	C(2)-C(1)-C(6)	τ74	C(1)-C(6)-C(5)-C(4)
ν18	C(5)-C(6)	δ47	C(1)-C(6)-C(5)	τ75	C(6)-C(5)-C(4)-C(3)
ν19	C(27)-C(23)	δ48	C(6)-C(5)-C(4)	τ76	C(4)-C(3)-C(12)-C(15)
ν20	C(4)-C(3)	δ49	C(4)-C(3)-C(12)	τ77	C(5)-C(4)-C3)-C(12)
ν21	C(5)-C(4)	δ50	C(5)-C(4)-C(3)	τ78	C(20)-C(21)-C(23)-C(27)
ν22	C(20)-C(21)	δ51	C(20)-C(21)-C(23)	τ79	C(25)-C(27)-C(23)-C(21)
ν23	C(21)-C(23)	δ52	C(21)-C(23)-C(27)	τ80	C(3)-C(12)-S(15)-S(16)
ν24	C(25)-C(27)	δ53	C(25)-C(27)-C(23)	τ81	C(12)-S(15)-S(16)-C(17)
ν25	C(3)-C(12)	δ54	C(17)-C(20)-C(22)	τ82	S(16)-C(17)-C(20)-C(21)
ν26	C(17)-C(20)	δ55	C(12)-S(15)-S(16)	τ83	S(15)-S(16)-C(17)-C(20)
ν27	S(15)-C(12)	δ56	S(16)-C(17)-C(20)	γ84	C(17)-C(21)-C(22)-C(20)
ν28	S(16)-C(17)	δ57	S(15)-S(16)-C(17)		
ν29	S(15)-S(16)				

**Table 3:** Assignment of high frequency normal mode of DBDS-1.

Normal Mode	Scaled Frequency (cm${}^{-1}$)	Vibration Assignment (>10%)
#1	3077.87	%14ν(1) + %11ν(3) + %25ν(4) + %34ν(5)
#2	3077.32	%40ν(10) + %20ν(12) + %16ν(14)
#3	3071.02	%38ν(10) + %31ν(13) + %24ν(14)
#4	3068.73	%27ν(1) + %41ν(3) + %15ν(4)
#5	3061.73	%23ν(1) + %22ν(2) + %24ν(3) + %23ν(5)
#6	3061.38	%23ν(11) + %22ν(12) + %25ν(13) + %21ν(14)
#7	3053.17	%22ν(2) + %20ν(3) + %42ν(4) + %16ν(5)
#8	3051.51	%36ν(11) + %40ν(12) + %14ν(14)
#9	3046.70	%33ν(1) + %47ν(2) + %12ν(5)
#10	3045.33	%33ν(11) + %14ν(12) + %30ν(13) + %21ν(14)
#11	3024.71	%88ν(8) + %11ν(9)
#12	3007.35	%77ν(6) + %23ν(7)
#13	2946.40	%22ν(6) + %77ν(7)
#14	2942.76	%11ν(8) + %88ν(9)

**Table 4:** Assignment of middle frequency normal mode of DBDS-1.

Normal Mode	Scaled Frequency (cm${}^{-1}$)	Vibration Assignment (>10%)
#15	1590.90	%30ν(16) + %10ν(19)
#16	1588.63	%30ν(15) + %11ν(18)
#17	1571.78	%28ν(19) + %21ν(22)
#18	1569.58	%27ν(18) + %23ν(20)
#19	1476.26	%16δ(41) + %15δ(42) + %16δ(43) + %18δ(44)
#20	1474.92	%19δ(32) + %15δ(33) + %15δ(34) + %16δ(35)
#21	1437.59	%11δ(40) + %20δ(45)
#22	1434.55	%10ν(21) + %10δ(35) + %24δ(36)
#23	1420.17	%53δ(40) + %12τ(66)
#24	1415.30	%60δ(38) + %12τ(63) + %16τ(64)
#25	1313.79	%27δ(41) + %24δ(42) + %11δ(44) + %10δ(45)
#26	1311.88	%11δ(32) + %25δ(33) + %26δ(34) + %10δ(35) + %10δ(36)
#27	1296.69	%20ν(16) + %22ν(22) + %17ν(23) + %16ν(24)
#28	1295.12	%18ν(15) + %17ν(17) + %24ν(20) + %16ν(21)
#29	1225.70	%12δ(39) + %18δ(40) + %12τ(65) + %16τ(66)
#30	1220.57	%20δ(38) + %11τ(63) + %14τ(64)
#31	1190.12	%22ν(25) + %14ν(26)
#32	1188.81	%12ν(25) + %22ν(26)
#33	1162.78	%15δ(41) + %16δ(42) + %14δ(43) + %11δ(44)
#34	1160.99	%13δ(32) + %16δ(33) + %15δ(34) + %13δ(35)
#35	1141.18	%18δ(32) + %19δ(35) + %38δ(36)
#36	1140.39	%19δ(43) + %19δ(44) + %37δ(45)
#37	1126.57	%33δ(39) + %10τ(65) + %34τ(66)
#38	1123.89	%35δ(37) + %10τ(63) + %31τ(64)
#39	1062.59	%13ν(16) + %15ν(23) + %10δ(39) + %10δ(42) + %13δ(45)
#40	1060.74	%13ν(15) + %15ν(21) + %12δ(36) + %11δ(37)
#41	1015.83	%19ν(19) + %24ν(24) + %13δ(30) + %11δ(43) + %10δ(44)
#42	1014.97	%24ν(17) + %21ν(18) + %11δ(35) + %15δ(46)
#43	985.97	%10ν(19) + %11ν(24) + %26δ(30) + %13δ(52) + %18δ(53)
#44	985.52	%11ν(17) + %10ν(18) + %25δ(46) + %19δ(47) + %13δ(48)
#45	967.00	%22τ(58) + %15τ(61) + %26τ(62) + %16τ(72)
#46	964.61	%20τ(69) + %18τ(70) + %30τ(71) + %10τ(73)
#47	948.26	%17τ(58) + %16τ(59) + %15τ(60) + %26τ(61)
#48	943.31	%19τ(67) + %17τ(68) + %23τ(69) + %26τ(70)
#49	897.82	%21τ(67) + %19τ(68) + %24τ(71)
#50	894.49	%20τ(59) + %23τ(60) + %24τ(62)
#51	864.65	%14δ(39) + %11τ(63) + %20τ(65)
#52	862.53	%17δ(37) + %20τ(63) + %11τ(65)
#53	826.45	%22τ(58) + %22τ(59) + %23τ(60) + %20τ(61)
#54	823.51	%21τ(67) + %24τ(68) + %21τ(69) + %21τ(70)
#55	797.93	%19ν(26) + %10δ(52) + %30δ(53)
#56	794.54	%18ν(25) + %30δ(47)
#57	750.92	%17τ(58) + %15τ(61) + %14τ(62) + %21τ(75)
#58	745.18	%14τ(69) + %13τ(70) + %11τ(71) + %20τ(78) + %13γ(84)
#59	687.46	%10τ(59) + %10τ(60) + %15τ(62) + %10τ(72) + %19τ(75)
#60	685.91	%10τ(68) + %14τ(71) + %25τ(78)
#61	634.47	%50ν(27)
#62	628.24	%41ν(28)
#63	614.53	%23δ(46) + %33δ(48) + %19δ(50)
#64	613.15	%13ν(28) + %20δ(30) + %27δ(52)
#65	558.03	%20δ(51) + %14δ(56)
#66	551.85	%16δ(31) + %19δ(50)
#67	468.56	%11ν(27) + %17ν(29) + %22τ(74)
#68	462.98	%30ν(29) + %14γ(84)
#69	454.94	%51ν(29)

**Table 5:** Comparison of experimental and theoretical Raman spectrum.

Experimental Raman Peak (cm${}^{-1}$)	Corresponding Calculated Peak (cm${}^{-1}$)	Mode Identification	Deviation (cm${}^{-1}$)
467	456	#69	11
481	467	#67 + #68	14
504	-	Overtone of low frequency vibration	-
567	552	#65 + #66	15
616	615	#63 + #64	1
659	630	#61 + #62 (DBDS-1)	29
701	666	#61 + #62 (DBDS-2)	35
772	760	#57 (DBDS-2)	12
811	796	#55 + #56	15
860	824	#53 + #54	36
920	864	#51 + #52	56
1002	985	#43 + #44	17
1033	1015	#41 + #42	18
1074	1062	#39 + #40	12
-	1125	#37 + #38	-
1143	1140	#35 + #36	3
1162	1162	#33 + #34	0
1202	1189	#31 + #32	13
1234	1225	#29 + #30	9
1295	1296	#27 + #28	−1
1324	1312	#25 + #26	12
1338	-	Double frequency band of 659	-
1411	1416	#23 + #24	−5
1452	1432	#21 + #22	20
1463	1475	#19 + #20	−12
1495	-	Combination of 1002 and 481 under Fermi resonance	-
1526	-	Combination of 1002 and 481 under Fermi resonance	-
1584	1571	#17 + #18	13
1600	1590	#15 + #16	10
2908	2946	#13 + #14(DBDS-1)	−38
2964	2972	#13 (DBDS-2)	−8
3002	3008	#12	−6
3028	3025	#11 (DBDS-1)	3
3042	3053	#7 + #8	−11
3049	3062	#5 + #6	−13
3068	3077	#1 + #2	−9

**Table 6:** Comparison of experimental and theoretical IR spectrum.

Experimental IR Peak (cm${}^{-1}$)	Corresponding Calculated Peak (cm${}^{-1}$)	Mode Identification	Deviation (cm${}^{-1}$)
467	467	#67 + #68	0
505	-	Overtone of low frequency vibration	-
563	555	#65 + #66	8
569	-	Overtone of low frequency vibration	-
617	615	#63 + #64	2
658	633	#61 + #62	25
695	687	#59 + #60	8
757	751	#57 + #58 (DBDS-1)	6
766	758	#57 (DBDS-2)	8
803	796	#55 + #56	7
816	826	#53 + #54	−10
864	864	#51 + #52	0
912	895	#49 + #50	17
971	947	#47 + #48	24
1001	986	#43 + #44	15
1028	1015	#41 + #42	13
1068	1062	#39 + #40	6
1112	1126	#37 + #38	−14
1139	1162	#33 + #34	−23
1181	1190	#31 + #32	−9
1198	-	Combination of 695 and 563	-
1223	1223	#29 + #30	0
1291	1296	#27 + #28	−5
1336	1313	#25 + #26	23
1409	1416	#23 + #24	−7
1452	1436	#21 + #22	16
1494	1475	#19 + #20	19
1582	1570	#17 + #18	12
1599	1588	#15 + #16	11
2909	2946	#13 + #14 (DBDS-1)	−37
2964	2972	#13 (DBDS-2)	−8
3006	3007	#12	−1
3026	3025	#11 (DBDS-1)	1
3043	3047	#9 + #10	−4
3052	3062	#5 + #6	−10
3068	3070	#3 + #4	−2
3083	3077	#1 + #2	6

**Table 7:** The characteristic vibration frequency of methylene groups in different molecular.

Conformation	Stretching Vibration (cm${}^{-1}$)		Bending Vibration (cm${}^{-1}$)			
	Asymmetric ($\nu_{s}$)	Symmetric ($\nu_{\mathrm{as}}$)	Scissoring (δ)	Wagging (ω)	Twisting (τ)	Rocking (ρ)
DBDS-1	3007/3025	2943/2946	1415/1420	1221/1226	1124/1127	863/865
DBDS-2	3007/3041	2947/2971	1417/1431	1220/1227	1112/1122	878/894
DBS	2990/2991	2944	1412/1416	1223/1237	1130/1155	878/918
BiBz-1	2956/2968	2919/2924	1438/1443	1279/1324	1126/1170	921/977
BiBz-2	2950/2972	2920/2929	1436/1452	1251/1316	1122/1248	748/964

## Data Availability

The data presented in this study are available on request from the corresponding author.

## Supplementary Materials

The following supporting information can be downloaded at: https://www.mdpi.com/article/10.3390/ijms23041958/s1.

## References

[1] Scatiggio F., Tumiatti V., Maina R., Tumiatti M., Pompili M., Bartnikas R. (2009). Corrosive sulfur induced failures in oil-filled electrical power transformers and shunt reactors. IEEE Trans. Power Deliv..

[2] Scatiggio F., Tumiatti V., Maina R., Tumiatti M., Pompili M., Bartnkas R. (2007). Corrosive sulfur in insulating oils: Its detection and correlated power apparatus failures. IEEE Trans. Power Deliv..

[3] Dahlund M. (2009). Copper Sulphide in Transformer Insulation.

[4] Yuan Y., Gao X., Zhou J., Liu G., Kuang X., Yang L., Liao R. (2021). A review: Research on corrosive sulphur in electrical power equipment. High Volt..

[5] Cong H., Pan H., Qian D., Zhao H., Li Q. (2020). Reviews on sulphur corrosion phenomenon of the oil–paper insulating system in mineral oil transformer. High Volt..

[6] Toyama S., Tanimura J., Yamada N., Nagao E., Amimoto T. (2009). Highly sensitive detection method of dibenzyl disulfide and the elucidation of the mechanism. IEEE Trans. Dielectr. Electr. Insul..

[7] Kamishima S., Ito T., Morishima Y. (2012). Change in corrosivity of insulating oil caused by oxidative deterioration of the oil. IEEE Trans. Dielectr. Electr. Insul..

[8] Lukic J.M., Milosavljevic S.B., Orlovic A.M. (2010). Degradation of the insulating system of power transformers by copper sulfide deposition: Influence of oil oxidation and presence of metal passivator. Ind. Eng. Chem. Res..

[9] Cong H., Pan H., Hu X., Zhang M., Li Q. (2021). Micro-mechanism study on synergistic degradation of the oil-paper insulation with dibenzyl disulfide, hexadecyl mercaptan and benzothiophene. High Volt..

[10] Jaber A., Mehanna N., Oweimreen G., Abulkibash A. (2016). The effect of DBDS, DBPC, BTA and DBP combinations on the corrosion of copper immersed in mineral transformer oil. IEEE Trans. Dielectr. Electr. Insul..

[11] Tumiatti V., Roggero C., Tumiatti M., Di Carlo S., Maina R., Kapila S. (2012). IEC 62697-2012: State of the art methods for quantification of DBDS and other corrosive sulfur compounds in unused and used insulating liquids. IEEE Trans. Dielectr. Electr. Insul..

[12] Du D., Tang C., Zhang J., Hu D. (2020). Effects of hydrogen sulfide on the mechanical and thermal properties of cellulose insulation paper: A molecular dynamics simulation. Mater. Chem. Phys..

[13] Naicker S.A., Moodley M. (2021). A computational study of the adsorption of corrosive sulphur on Ag surfaces. J. Mater. Sci..

[14] Zhang Y., Katayama Y., Tatara R., Giordano L., Yu Y., Fraggedakis D., Sun J.G., Maglia F., Jung R., Bazant M.Z. (2020). Revealing electrolyte oxidation via carbonate dehydrogenation on Ni-based oxides in Li-ion batteries by in situ Fourier transform infrared spectroscopy. Energy Environ. Sci..

[15] Jianxin W., Weigen C., Zhixian Z., Fu W., Feng Z., Ruimin S., Yingying W., Shoufei G. (2021). Fiber-enhanced Raman spectroscopy for highly sensitive H_2_ and SO_2_ sensing with a hollow-core anti-resonant fiber. Opt. Express.

[16] Song R., Chen W., Yang D., Shi H., Zhang R., Wang Z. (2021). Aging Assessment of Oil–Paper Insulation Based on Visional Recognition of the Dimensional Expanded Raman Spectra. IEEE Trans. Instrum. Meas..

[17] Inaoka S., Iwata K., Saha S. (2020). Towards the critical understanding of selected vibrational features in biologically important dicyano aromatic conjugated molecules: Importance of electron donating/withdrawal groups and geometry associated with dicyano group. Spectrochim. Acta Part A Mol. Biomol. Spectrosc..

[18] Wu Y., Lan H., Ning W., Chen F., Yan G., Cai K. (2021). Structural dynamics and vibrational feature of *N*-Acetyl-D-glucosamine in aqueous solution. Spectrochim. Acta Part A Mol. Biomol. Spectrosc..

[19] Muthunatesan S., Ragavendran V. (2015). A study of vibrational spectra and investigations of charge transfer and chemical bonding features of 2-chloro benzimidazole based on DFT computations. Spectrochim. Acta Part A Mol. Biomol. Spectrosc..

[20] Balachandran V., Santhi G., Karpagam V., Rastogi V. (2014). Structural features of the 2-amino-5-nitrobenzophenone by means of vibrational spectroscopy HF and DFT, first order hyperpolarizability, NBO, HOMO–LUMO and theromodynamic properties. Spectrochim. Acta Part A Mol. Biomol. Spectrosc..

[21] Sert Y., El-Emam A.A., Al-Abdullah E.S., Al-Tamimi A.M.S., Çırak Ç., Ucun F. (2014). Use of vibrational spectroscopy to study 4-benzyl-3-(thiophen-2-yl)-4, 5-dihydro-1H-1, 2, 4-triazole-5-thione: A combined theoretical and experimental approach. Spectrochim. Acta Part A Mol. Biomol. Spectrosc..

[22] Kose E., Bardak F., Atac A., Karabacak M., Cipiloglu M. (2013). Determination of structural and vibrational spectroscopic features of neutral and anion forms of dinicotinic acid by using NMR, infrared and Raman experimental methods combined with DFT and HF. Spectrochim. Acta Part A Mol. Biomol. Spectrosc..

[23] Chen W., Gu Z., Zou J., Wan F., Xiang Y. (2016). Analysis of furfural dissolved in transformer oil based on confocal laser Raman spectroscopy. IEEE Trans. Dielectr. Electr. Insul..

[24] Lu T. Molclus Program. Version 1.9.9.5. http://www.keinsci.com/research/molclus.html.

[25] Frisch M.J., Trucks G.W., Schlegel H.B., Scuseria G.E., Robb M.A., Cheeseman J.R., Scalmani G., Barone V., Petersson G.A., Nakatsuji H. (2016). Gaussian 16, Revision A.03.

[26] Stewart J.J. (2013). Optimization of parameters for semiempirical methods VI: More modifications to the NDDO approximations and re-optimization of parameters. J. Mol. Model..

[27] Stephens P.J., Devlin F.J., Chabalowski C.F., Frisch M.J. (1994). Ab initio calculation of vibrational absorption and circular dichroism spectra using density functional force fields. J. Phys. Chem..

[28] Grimme S., Antony J., Ehrlich S., Krieg H. (2010). A consistent and accurate ab initio parametrization of density functional dispersion correction (DFT-D) for the 94 elements H-Pu. J. Chem. Phys..

[29] Grimme S., Ehrlich S., Goerigk L. (2011). Effect of the damping function in dispersion corrected density functional theory. J. Comput. Chem..

[30] Hariharan P., Pople J. (1974). Accuracy of AH n equilibrium geometries by single determinant molecular orbital theory. Mol. Phys..

[31] Petersson A., Bennett A., Tensfeldt T.G., Al-Laham M.A., Shirley W.A., Mantzaris J. (1988). A complete basis set model chemistry. I. The total energies of closed-shell atoms and hydrides of the first-row elements. J. Chem. Phys..

[32] Santra G., Sylvetsky N., Martin J.M. (2019). Minimally empirical double-hybrid functionals trained against the GMTKN55 database: revDSD-PBEP86-D4, revDOD-PBE-D4, and DOD-SCAN-D4. J. Phys. Chem. A.

[33] Papajak E., Zheng J., Xu X., Leverentz H.R., Truhlar D.G. (2011). Perspectives on basis sets beautiful: Seasonal plantings of diffuse basis functions. J. Chem. Theory Comput..

[34] Lu T., Chen Q. (2021). Shermo: A general code for calculating molecular thermochemistry properties. Comput. Theor. Chem..

[35] Humphrey W., Dalke A., Schulten K. (1996). VMD—Visual Molecular Dynamics. J. Mol. Graph..

[36] Zhang J., Lu T. (2021). Efficient evaluation of electrostatic potential with computerized optimized code. Phys. Chem. Chem. Phys..

[37] Johnson E.R., Keinan S., Mori-Sánchez P., Contreras-García J., Cohen A.J., Yang W. (2010). Revealing noncovalent interactions. J. Am. Chem. Soc..

[38] Lu T., Chen F. (2012). Multiwfn: A multifunctional wavefunction analyzer. J. Comput. Chem..

[39] Lu T., Chen Q. (2021). Interaction Region Indicator: A Simple Real Space Function Clearly Revealing Both Chemical Bonds and Weak Interactions. Chem.-Methods.

[40] Jamróz M.H. (2013). Vibrational energy distribution analysis (VEDA): Scopes and limitations. Spectrochim. Acta Part A Mol. Biomol. Spectrosc..

[41] Kashinski D., Chase G., Nelson R., Di Nallo O., Scales A., VanderLey D., Byrd E. (2017). Harmonic vibrational frequencies: Approximate global scaling factors for TPSS, M06, and M11 functional families using several common basis sets. J. Phys. Chem. A.

[42] Liu Z., Lu T., Chen Q. (2021). Vibrational Spectra and Molecular Vibrational Behaviors of All-Carboatomic Rings, cyclo [18] carbon and Its Analogues. Chem.-Asian J..

[43] Halls M.D., Velkovski J., Schlegel H.B. (2001). Harmonic frequency scaling factors for Hartree-Fock, S-VWN, B-LYP, B3-LYP, B3-PW91 and MP2 with the Sadlej pVTZ electric property basis set. Theor. Chem. Accounts.

