# Supplementary material for: Vibrational Spectra and Molecular Vibrational Behaviors of Dibenzyl Disulfide, Dibenzyl Sulphide and Bibenzyl
Source: Int J Mol Sci. 2022 Feb 10;23(4):1958. doi: 10.3390/ijms23041958 (PMC8874789; doi:10.3390/ijms23041958)
Supplement: Supplementary file 1 [file ijms-23-01958-s001.zip › ijms-1584064-SI.pdf]

Supplementary Material

4. Vibration analysis and vibrational spectra

4.1. PED based vibrational assignment

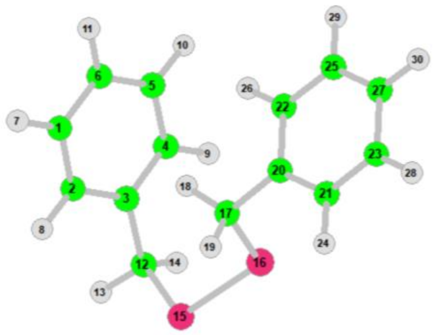

Fig. S1. The atomic number of DBDS-2 in VEDA4

Table. S1. Internal coordinates and related atoms in DBDS-2

Stretching	Related atoms	Bending	Related atoms
ν1	C(1)-H(7)	δ30	C(21)-C(23)-C(27)
ν2	C(2)-H(8)	δ31	C(3)-C(12)-S(15)
ν3	C(4)-H(9)	δ32	H(7)-C(1)-C(2)
ν4	C(5)-H(10)	δ33	H(8)-C(2)-C(1)
ν5	C(6)-H(11)	δ34	H(9)-C(4)-C(5)
ν6	C(12)-H(13)	δ35	H(10)-C(5)-C(6)
ν7	C(12)-H(14)	δ36	H(11)-C(6)-C(1)
ν8	C(17)-H(18)	δ37	H(13)-C(12)-S(15)
ν9	C(17)-H(19)	δ38	H(14)-C(12)-H(13)
ν10	C(21)-H(24)	δ39	H(18)-C(17)-S(16)
ν11	C(22)-H(26)	δ40	H(19)-C(17)-H(18)
ν12	C(23)-H(28)	δ41	H(24)-C(21)-C(23)
ν13	C(25)-H(29)	δ42	H(26)-C(22)-C(25)
ν14	C(27)-H(30)	δ43	H(28)-C(23)-C(27)
ν15	C(2)-C(1)	δ44	H(29)-C(25)-C(27)
ν16	C(21)-C(23)	δ45	H(30)-C(27)-C(25)
ν17	C(1)-C(6)	δ46	C(2)-C(1)-C(6)
ν18	C(6)-C(5)	δ47	C(1)-C(6)-C(5)
ν19	C(22)-C(25)	δ48	C(6)-C(5)-C(4)
ν20	C(4)-C(3)	δ49	C(22)-C(25)-C(27)
ν21	C(5)-C(4)	δ50	C(4)-C(3)-C(12)

ν22	C(23)-C(27)	δ51	C(5)-C(4)-C(3)
ν23	C(27)-C(25)	δ52	C(23)-C(27)-C(25)
ν24	C(20)-C(22)	δ53	C(20)-C(22)-C(25)
ν25	C(3)-C(12)	δ54	C(17)-C(20)-C(22)
ν26	C(17)-C(20)	δ55	C(12)-S(15)-S(16)
ν27	S(15)-C(12)	δ56	S(16)-C(17)-C(20)
ν28	S(16)-C(17)	δ57	S(15)-S(16)-C(17)
ν29	S(15)-S(16)		

Torsion	Related atoms
τ58	H(7)-C(1)-C(2)-C(3)
τ59	H(8)-C(2)-C(1)-C(6)
τ60	H(9)-C(4)-C(5)-C(6)
τ61	H(10)-C(5)-C(6)-C(1)
τ62	H(11)-C(6)-C(1)-C(2)
τ63	H(13)-C(12)-S(15)-S(16)
τ64	H(14)-C(12)-S(15)-S(16)
τ65	H(18)-C(17)-S(16)-S(15)
τ66	H(19)-C(17)-S(16)-S(15)
τ67	H(24)-C(21)-C(23)-C(27)
τ68	H(26)-C(22)-C(25)-C(27)
τ69	H(28)-C(23)-C(27)-C(25)
τ70	H(29)-C(25)-C(27)-C(23)
τ71	H(30)-C(27)-C(25)-C(22)
τ72	C(2)-C(1)-C(6)-C(5)
τ73	C(21)-C(23)-C(27)-C(25)
τ74	C(1)-C(6)-C(5)-C(4)
τ75	C(6)-C(5)-C(4)-C(3)
τ76	C(4)-C(3)-C(12)-S(15)
τ77	C(5)-C(4)-C(3)-C(12)
τ78	C(23)-C(27)-C(25)-C(22)
τ79	C(20)-C(22)-C(25)-C(27)
τ80	C(3)-C(12)-S(15)-S(16)
τ81	C(12)-S(15)-S(16)-C(17)
τ82	S(16)-C(17)-C(20)-C(21)
τ83	S(15)-S(16)-C(17)-C(20)
γ84	C(17)-C(21)-C(22)-C(20)

Table. S2. Assignment of normal mode of DBDS-2.

Mode	Frequency	Vibration assignment (>10%)
#1	3077.9556	%20ν(1)+%17ν(3)+%22ν(4)+%35ν(5)
#2	3076.77952	%18ν(11)+%21ν(12)+%23ν(13)+%31ν(14)
#3	3070.44604	%25ν(1)+%50ν(3)
#4	3069.69412	%48ν(11)+%26ν(12)
#5	3062.7244	%24ν(1)+%19ν(2)+%17ν(3)+%11ν(4)+%28ν(5)

#6	3061.84716	%22ν(10)+%18ν(11)+%18ν(12)+%13ν(13)+%28ν(14)
#7	3052.77592	%30ν(2)+%12ν(3)+%44ν(4)+%13ν(5)
#8	3052.44816	%38ν(10)+%10ν(11)+%36ν(13)
#9	3047.48356	%27ν(1)+%42ν(2)+%12ν(4)+%15ν(5)
#10	3046.74128	%29ν(10)+%28ν(12)+%18ν(13)+%23ν(14)
#11	3041.44892	%78ν(8)+%21ν(9)
#12	3007.36188	%23ν(6)+%76ν(7)
#13	2971.53964	%21ν(8)+%79ν(9)
#14	2947.3818	%76ν(6)+%23ν(7)
#15	1589.85772	%29ν(16)
#16	1588.58524	%29ν(15)+%11ν(18)
#17	1570.115	%28ν(23)+%21ν(24)+%10δ(30)
#18	1569.27632	%27ν(18)+%23ν(20)
#19	1474.89108	%14δ(41)+%14δ(42)+%16δ(43)+%15δ(44)
#20	1474.5344	%18δ(32)+%14δ(33)+%14δ(34)+%15δ(35)
#21	1437.16012	%28δ(40)+%10δ(45)
#22	1434.42236	%15δ(36)
#23	1431.04836	%39δ(40)+%11τ(66)
#24	1417.31136	%56δ(38)+%12τ(63)+%16τ(64)
#25	1311.91724	%24δ(41)+%26δ(42)+%10δ(43)+%10δ(45)
#26	1311.46416	%10δ(32)+%24δ(33)+%25δ(34)
#27	1295.7124	%18ν(15)+%17ν(17)+%23ν(20)+%16ν(21)
#28	1294.88336	%17ν(16)+%17ν(19)+%18ν(22)+%23ν(24)
#29	1226.55504	%10ν(26)+%25δ(40)+%20τ(65)+%28τ(66)
#30	1220.2312	%10ν(25)+%29δ(38)+%14τ(63)+%20τ(64)
#31	1189.11328	%32ν(25)
#32	1187.12744	%33ν(26)
#33	1161.48504	%17δ(32)+%21δ(33)+%20δ(34)+%18δ(35)
#34	1160.897	%21δ(41)+%21δ(42)+%16δ(43)+%17δ(44)
#35	1141.12536	%16δ(36)+%11δ(43)+%10δ(44)+%22δ(45)
#36	1140.894	%10δ(32)+%11δ(35)+%22δ(36)+%17δ(45)
#37	1122.0478	%46δ(37)+%12τ(63)+%17τ(64)
#38	1111.84868	%39δ(39)+%10τ(65)+%27τ(66)
#39	1060.81452	%13ν(15)+%15ν(21)+%12δ(36)+%12δ(37)
#40	1057.86468	%12ν(16)+%13ν(19)+%17δ(39)+%11δ(45)
#41	1015.41976	%22ν(22)+%20ν(23)+%13δ(30)+%11δ(43)+%11δ(44)
#42	1014.61	%24ν(17)+%21ν(18)+%11δ(35)+%15δ(46)
#43	985.91172	%20δ(46)+%15δ(47)+%10δ(48)
#44	985.48756	%20δ(30)+%10δ(49)+%15δ(52)
#45	967.7596	%14τ(58)+%17τ(61)+%23τ(62)+%12τ(72)
#46	966.70884	%16τ(69)+%14τ(70)+%25τ(71)
#47	947.3228	%23τ(58)+%19τ(59)+%14τ(60)+%22τ(61)
#48	944.54648	%18τ(67)+%20τ(68)+%27τ(69)+%26τ(70)
#49	899.52768	%24τ(67)+%25τ(68)+%30τ(71)

#50	894.70768	%24τ(59)+%27τ(60)+%28τ(62)
#51	878.13652	%28δ(39)+%38τ(65)
#52	863.33912	%20δ(37)+%30τ(63)+%11τ(64)
#53	826.7264	%14τ(70)+%13τ(69)+%12τ(60)+%12τ(59)+%15τ(67)+%13τ(68)
#54	824.20072	%13τ(58)+%14τ(59)+%15τ(60)+%12τ(61)+%12τ(67)+%11τ(68)
#55	797.48828	%21ν(26)+%33δ(52)
#56	795.41568	%21ν(25)+%35δ(47)+%11δ(48)
#57	759.52596	%10ν(28)+%10τ(69)+%11τ(70)+%11τ(71)+%17τ(79)+%14γ(84)
#58	750.715	%18τ(58)+%15τ(61)+%14τ(62)+%22τ(75)
#59	687.36092	%12τ(67)+%11τ(68)+%18τ(71)+%14τ(73)+%11τ(78)+%27τ(79)
#60	685.7414	%13τ(59)+%13τ(60)+%19τ(62)+%13τ(72)+%12τ(74)+%25τ(75)
#61	665.2082	%44ν(28)+%20τ(79)
#62	631.59352	%57ν(27)
#63	615.42724	%23δ(30)+%34δ(49)+%20δ(53)
#64	614.07764	%23δ(46)+%32δ(48)+%19δ(51)
#65	555.37968	%12δ(31)+%15δ(51)
#66	551.78396	%15δ(53)
#67	472.63956	%13ν(28)+%25ν(29)+%16γ(84)
#68	466.66276	%13ν(27)+%10τ(72)+%25τ(74)
#69	457.28304	%69ν(29)

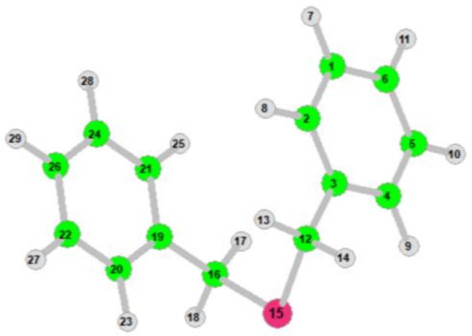

Fig. S2. The atomic number of DBS in VEDA4

Table. S3. Internal coordinates and related atoms in DBS

Stretching	Related atoms	Bending	Related atoms
ν1	C(1)-H(7)	δ29	δ29
ν2	C(2)-H(8)	δ30	δ30
ν3	C(4)-H(9)	δ31	δ31
ν4	C(5)-H(10)	δ32	δ32
ν5	C(6)-H(11)	δ33	δ33
ν6	C(12)-H(13)	δ34	δ34
ν7	C(12)-H(14)	δ35	δ35
ν8	C(16)-H(17)	δ36	δ36
ν9	C(16)-H(18)	δ37	δ37

ν10	C(20)-H(23)	δ38	δ38
ν11	C(21)-H(25)	δ39	δ39
ν12	C(22)-H(27)	δ40	δ40
ν13	C(24)-H(28)	δ41	δ41
ν14	C(26)-H(29)	δ42	δ42
ν15	C(1)-C(6)	δ43	δ43
ν16	C(2)-C(1)	δ44	δ44
ν17	C(4)-C(5)	δ45	δ45
ν18	C(20)-C(22)	δ46	δ46
ν19	C(26)-C(24)	δ47	δ47
ν20	C(3)-C(2)	δ48	δ48
ν21	C(6)-C(5)	δ49	δ49
ν22	C(21)-C(19)	δ50	δ50
ν23	C(22)-C(26)	δ51	δ51
ν24	C(24)-C(21)	δ52	δ52
ν25	C(12)-C(3)	δ53	δ53
ν26	C(19)-C(16)	δ54	δ54
ν27	S(15)-C(12)	δ55	δ55
ν28	S(15)-C(16)		

Torsion	Related atoms
τ56	H(7)-C(1)-C(2)-C(3)
τ57	H(8)-C(2)-C(1)-C(6)
τ58	H(9)-C(4)-C(5)-C(6)
τ59	H(10)-C(5)-C(6)-C(1)
τ60	H(11)-C(6)-C(1)-C(2)
τ61	H(13)-C(12)-S(15)-C(16)
τ62	H(14)-C(12)-S(15)-C(16)
τ63	H(17)-C(16)-S(15)-C(12)
τ64	H(18)-C(16)-S(15)-C(12)
τ65	H(23)-C(20)-C(22)-C(26)
τ66	H(25)-C(21)-C(24)-C(26)
τ67	H(27)-C(22)-C(26)-C(24)
τ68	H(28)-C(24)-C(26)-C(22)
τ69	H(29)-C(26)-C(24)-C(21)
τ70	C(2)-C(1)-C(6)-C(5)
τ71	C(4)-C(5)-C(6)-C(1)
τ72	C(20)-C(22)-C(26)-C(24)
τ73	C(26)-C(24)-C(21)-C(19)
τ74	C(3)-C(2)-C(1)-C(6)
τ75	C(21)-C(19)-C(16)-S(15)
τ76	C(22)-C(26)-C(24)-C(21)
τ77	C(24)-C(21)-C(19)-C(16)
τ78	C(19)-C(16)-S(15)-C(12)
τ79	S(15)-C(12)-C(3)-C(4)

τ80	C(16)-S(15)-C(12)-C(3)
γ81	C(12)-C(4)-C(2)-C(3)

Table. S4. Assignment of normal mode of DBS.

Mode	Frequency	Vibration assignment (>10%)
#1	3075.748	%10ν(1)+%10ν(4)+%16ν(5)+%13ν(12)+%12ν(13)+%21ν(14)
#2	3075.555	%12ν(1)+%14ν(4)+%21ν(5)+%11ν(12)+%17ν(14)
#3	3067.515	%13ν(12)+%13ν(2)+%12ν(13)+%11ν(3)+%13ν(10)+%18ν(11)
#4	3066.908	%14ν(1)+%13ν(2)+%16ν(3)+%13ν(4)+%11ν(10)+%11ν(11)
#5	3061.664	%13ν(2)+%13ν(3)+%14ν(5)+%20ν(10)+%19ν(11)+%21ν(14)
#6	3061.163	%20ν(2)+%18ν(3)+%21ν(5)+%12ν(10)+%13ν(11)+%14ν(14)
#7	3053.171	%15ν(13)+%12ν(3)+%13ν(4)+%14ν(10)+%12ν(1)+%14ν(12)
#8	3052.573	%13ν(12)+%13ν(2)+%13ν(3)+%16ν(4)+%12ν(10)+%12ν(1)
#9	3046.51	%12ν(1)+%10ν(4)+%11ν(5)+%15ν(12)+%17ν(13)+%17ν(14)
#10	3046.307	%18ν(1)+%14ν(4)+%16ν(5)+%10ν(12)+%12ν(13)+%11ν(14)
#11	2990.685	%64ν(8)+%35ν(9)
#12	2990.232	%64ν(6)+%35ν(7)
#13	2944.152	%35ν(8)+%63ν(9)
#14	2943.709	%34ν(6)+%64ν(7)
#15	1589.366	%14ν(17)+%16ν(18)
#16	1588.749	%16ν(17)+%14ν(18)
#17	1570.366	%15ν(15)+%13ν(19)+%12ν(20)+%10ν(22)
#18	1570.038	%13ν(15)+%16ν(19)+%10ν(20)+%12ν(22)
#19	1474.679	%10δ(41)+%11δ(42)
#20	1474.409	%10δ(31)+%10δ(32)+%11δ(34)
#21	1436.08	%14δ(44)
#22	1435.512	%14δ(35)
#23	1415.808	%22δ(37)+%34δ(39)
#24	1412.173	%41δ(37)+%28δ(39)
#25	1312.881	%12δ(32)+%10δ(33)+%14δ(40)+%16δ(41)
#26	1311.618	%16δ(32)+%14δ(33)+%11δ(40)+%12δ(41)
#27	1295.433	%11ν(20)+%12ν(22)
#28	1294.266	%10ν(17)+%12ν(20)+%11ν(22)
#29	1237.294	%19τ(62)+%19τ(64)
#30	1223.017	-
#31	1181.421	%16ν(25)+%22ν(26)
#32	1178.403	%21ν(25)+%16ν(26)
#33	1161.928	%10δ(33)+%13δ(40)+%11δ(41)+%10δ(43)
#34	1160.752	%10δ(31)+%11δ(32)+%13δ(33)+%10δ(40)
#35	1154.833	%24δ(36)+%23δ(38)+%12τ(62)+%12τ(64)
#36	1140.181	%18δ(35)+%10δ(43)+%19δ(44)
#37	1139.949	%10δ(31)+%19δ(35)+%17δ(44)
#38	1130.049	%14δ(36)+%15δ(38)+%20τ(62)+%20τ(64)
#39	1064.632	-

#40	1062.636	-
#41	1015.641	%11ν(15)+%10ν(19)+%13ν(21)+%12ν(23)
#42	1015.217	%10ν(15)+%11ν(19)+%12ν(21)+%13ν(23)
#43	985.5454	%19δ(47)+%10δ(48)+%14δ(51)
#44	985.4297	%19δ(29)+%14δ(45)
#45	966.1786	%10τ(56)+%10τ(59)+%13τ(60)+%12τ(67)+%11τ(68)+%17τ(69)
#46	965.6292	%13τ(56)+%13τ(59)+%18τ(60)+%10τ(67)+%13τ(69)
#47	947.612	%12τ(65)+%14τ(66)+%16τ(67)+%16τ(68)
#48	946.2142	%14τ(56)+%12τ(57)+%12τ(58)+%15τ(59)+%10τ(68)
#49	917.9883	-
#50	896.7803	%13τ(57)+%12τ(58)+%15τ(60)+%13τ(65)+%14τ(66)+%16τ(69)
#51	878.069	%12τ(60)+%10τ(69)
#52	828.7026	%13τ(67)+%13τ(68)+%12τ(58)+%11τ(59)+%14τ(65)+%12τ(66)
#53	826.3312	%10τ(56)+%10τ(58)+%10τ(65)
#54	822.7836	-
#55	793.4106	%15ν(26)+%12δ(45)+%23δ(51)
#56	793.1696	%14ν(25)+%23δ(45)+%12δ(51)
#57	748.6328	%16τ(73)
#58	741.7594	%16τ(73)
#59	691.082	%10ν(28)+%13τ(69)
#60	690.0312	%11τ(60)+%11τ(73)+%18τ(74)
#61	681.413	%11ν(27)+%10ν(28)+%14τ(73)+%15τ(74)
#62	657.3709	%26ν(27)+%27ν(28)
#63	613.4125	%10δ(29)+%13δ(46)+%16δ(47)+%21δ(48)+%11δ(52)
#64	613.0269	%16δ(29)+%20δ(46)+%10δ(47)+%13δ(48)+%11δ(49)
#65	561.8674	%11δ(49)+%10δ(52)
#66	551.3502	%12δ(49)+%11δ(52)
#67	462.6332	%14τ(76)+%12γ(81)
#68	458.9411	%12τ(76)+%14γ(81)

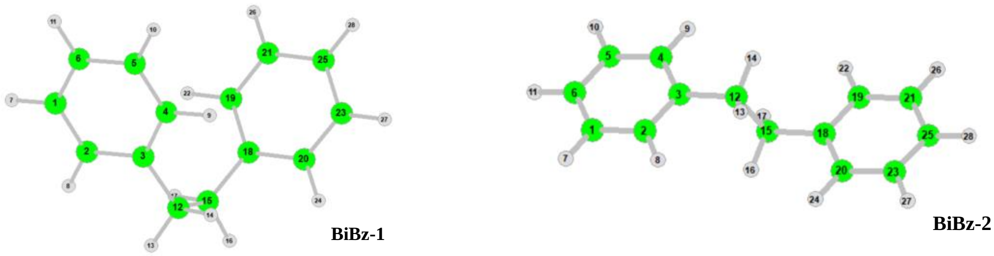

Fig. S3. The atomic number of BiBz-1 and BiBz-2 in VEDA4 (both are the same).

Table. S5. Internal coordinates and related atoms in BiBz-1 and BiBz-2 (both are the same).

Stretching	Related atoms	Bending	Related atoms
ν1	C(1)-H(7)	δ28	C(19)-C(21)-C(25)
ν2	C(2)-H(8)	δ29	C(3)-C(12)-C(15)
ν3	C(4)-H(9)	δ30	H(7)-C(1)-C(2)

ν4	C(5)-H(10)	δ31	H(8)-C(2)-C(1)
ν5	C(6)-H(11)	δ32	H(9)-C(4)-C(5)
ν6	C(12)-H(13)	δ33	H(10)-C(5)-C(6)
ν7	C(12)-H(14)	δ34	H(11)-C(6)-C(1)
ν8	C(15)-H(16)	δ35	H(13)-C(12)-C(3)
ν9	C(15)-H(17)	δ36	H(14)-C(12)-H(13)
ν10	C(19)-H(22)	δ37	H(16)-C(15)-C(12)
ν11	C(20)-H(24)	δ38	H(17)-C(15)-H(16)
ν12	C(21)-H(26)	δ39	H(22)-C(19)-C(21)
ν13	C(23)-H(27)	δ40	H(24)-C(20)-C(23)
ν14	C(25)-H(28)	δ41	H(26)-C(21)-C(25)
ν15	C(1)-C(6)	δ42	H(27)-C(23)-C(25)
ν16	C(2)-C(1)	δ43	H(28)-C(25)-C(23)
ν17	C(5)-C(4)	δ44	C(1)-C(6)-C(5)
ν18	C(6)-C(5)	δ45	C(2)-C(1)-C(6)
ν19	C(19)-C(21)	δ46	C(5)-C(4)-C(3)
ν20	C(20)-C(23)	δ47	C(6)-C(5)-C(4)
ν21	C(21)-C(25)	δ48	C(20)-C(23)-C(25)
ν22	C(25)-C(23)	δ49	C(21)-C(25)-C(23)
ν23	C(4)-C(3)	δ50	C(4)-C(3)-C(12)
ν24	C(18)-C(20)	δ51	C(18)-C(20)-C(23)
ν25	C(3)-C(12)	δ52	C(15)-C(18)-C(20)
ν26	C(15)-C(18)	δ53	C(12)-C(15)-C(18)
ν27	C(12)-C(15)		

Torsion	Related atoms
τ54	H(7)-C(1)-C(2)-C(3)
τ55	H(8)-C(2)-C(1)-C(6)
τ56	H(9)-C(4)-C(5)-C(6)
τ57	H(10)-C(5)-C(6)-C(1)
τ58	H(11)-C(6)-C(1)-C(2)
τ59	H(13)-C(12)-C(3)-C(4)
τ60	H(14)-C(12)-C(3)-C(4)
τ61	H(16)-C(15)-C(18)-C(19)
τ62	H(17)-C(15)-C(18)-C(19)
τ63	H(22)-C(19)-C(21)-C(25)
τ64	H(24)-C(20)-C(23)-C(25)
τ65	H(26)-C(21)-C(25)-C(23)
τ66	H(27)-C(23)-C(25)-C(21)
τ67	H(28)-C(25)-C(23)-C(20)
τ68	C(1)-C(6)-C(5)-C(4)
τ69	C(2)-C(1)-C(6)-C(5)
τ70	C(5)-C(4)-C(3)-C(12)
τ71	C(6)-C(5)-C(4)-C(3)
τ72	C(19)-C(21)-C(25)-C(23)

τ73	C(21)-C(25)-C(23)-C(20)
τ74	C(4)-C(3)-C(12)-C(15)
τ75	C(18)-C(20)-C(23)-C(25)
τ76	C(3)-C(12)-C(15)-C(18)
τ77	C(12)-C(15)-C(18)-C(19)
γ78	C(15)-C(19)-C(20)-C(18)

Table. S6. Assignment of normal mode of BiBz-1.

Mode	Frequency	Vibration assignment (>10%)
#1	3074.11888	%15ν(4)+%20ν(5)+%16ν(12)+%20ν(14)
#2	3074.04176	%16ν(4)+%20ν(5)+%15ν(12)+%19ν(14)
#3	3063.87156	%19ν(1)+%19ν(3)+%12ν(4)+%14ν(10)+%14ν(13)
#4	3063.76552	%14ν(1)+%14ν(3)+%10ν(4)+%19ν(10)+%13ν(12)+%19ν(13)
#5	3055.81252	%14ν(1)+%16ν(3)+%12ν(5)+%15ν(10)+%13ν(13)+%11ν(14)
#6	3055.639	%13ν(1)+%16ν(3)+%11ν(5)+%17ν(10)+%14ν(13)+%12ν(14)
#7	3047.09796	%11ν(3)+%18ν(4)+%11ν(5)+%13ν(10)+%22ν(12)+%13ν(14)
#8	3047.04012	%13ν(3)+%22ν(4)+%13ν(5)+%11ν(10)+%18ν(12)+%11ν(14)
#9	3036.83136	%18ν(1)+%71ν(2)
#10	3036.83136	%71ν(11)+%18ν(13)
#11	2968.4452	%15ν(6)+%35ν(7)+%15ν(8)+%35ν(9)
#12	2955.74932	%29ν(6)+%21ν(7)+%29ν(8)+%21ν(9)
#13	2924.52536	%21ν(6)+%29ν(7)+%21ν(8)+%29ν(9)
#14	2919.40652	%35ν(6)+%15ν(7)+%35ν(8)+%15ν(9)
#15	1591.8532	%16ν(16)+%14ν(20)
#16	1590.81208	%13ν(16)+%16ν(20)
#17	1570.66448	%17ν(18)+%11ν(21)+%13ν(22)
#18	1570.2114	%11ν(18)+%17ν(21)+%12ν(23)
#19	1476.40456	%10δ(30)
#20	1474.8236	-
#21	1443.50324	%35δ(36)+%37δ(38)
#22	1437.81564	%46δ(36)+%42δ(38)
#23	1434.06568	%15δ(34)+%11δ(43)
#24	1433.57404	%10δ(34)+%14δ(43)
#25	1323.83228	%12δ(35)+%19δ(37)+%17τ(59)
#26	1318.11576	%11τ(60)+%11τ(62)
#27	1311.60876	%10δ(31)+%13δ(32)+%14δ(39)+%11δ(40)
#28	1300.3878	-
#29	1294.652	%10ν(19)+%10ν(20)+%11ν(22)+%11ν(23)
#30	1278.6496	%18τ(60)+%18τ(62)
#31	1196.806	%13ν(25)+%13ν(26)
#32	1180.8036	%15ν(25)+%15ν(26)
#33	1169.61156	%18δ(35)+%24δ(37)+%10τ(61)
#34	1161.0898	%15δ(30)+%16δ(31)+%18δ(32)+%17δ(33)
#35	1160.86808	%17δ(39)+%16δ(40)+%17δ(41)+%14δ(42)

#36	1139.94928	%11δ(30)+%21δ(34)+%18δ(43)
#37	1139.50584	%17δ(34)+%10δ(41)+%11δ(42)+%21δ(43)
#38	1125.58568	%15δ(35)+%15δ(37)
#39	1071.76556	-
#40	1056.57292	-
#41	1016.28736	%13ν(15)+%11ν(18)+%10ν(24)
#42	1015.2366	%11ν(15)+%12ν(21)+%14ν(24)
#43	986.13344	%10δ(28)+%13δ(44)+%17δ(45)
#44	986.00812	%18δ(28)+%10δ(45)+%13δ(51)
#45	976.79228	%22ν(27)+%11δ(35)
#46	963.65296	%12τ(57)+%14τ(58)+%11τ(65)+%12τ(67)
#47	962.86248	%11τ(57)+%13τ(58)+%12τ(65)+%16τ(67)
#48	942.5028	%15τ(54)+%10τ(55)+%12τ(56)+%12τ(57)+%12τ(66)
#49	941.96296	%10τ(54)+%11τ(63)+%10τ(64)+%13τ(65)+%18τ(66)
#50	921.1502	-
#51	917.18816	%39ν(27)+%10τ(59)
#52	886.70648	%13τ(55)+%13τ(56)+%16τ(58)+%13τ(63)+%13τ(64)+%17τ(67)
#53	872.62244	%13τ(58)+%13τ(67)
#54	825.38644	%15τ(65)+%14τ(66)+%11τ(55)+%10τ(57)+%14τ(63)+%16τ(64)
#55	824.59596	%15τ(54)+%15τ(55)+%14τ(56)+%14τ(57)+%11τ(66)+%11τ(64)
#56	758.5234	%10ν(25)+%10ν(26)+%19δ(44)+%19δ(51)
#57	753.125	%11δ(44)+%12δ(51)
#58	742.24144	%12τ(71)+%12τ(74)
#59	733.7486	%11τ(74)
#60	690.16616	%11τ(67)+%10τ(71)+%16τ(74)
#61	689.46244	%11τ(58)+%12τ(71)+%13τ(74)
#62	616.16952	%10δ(28)+%14δ(45)+%12δ(46)+%20δ(47)+%16δ(48)
#63	615.8032	%12δ(28)+%12δ(45)+%10δ(46)+%18δ(47)+%19δ(48)
#64	574.40904	%13δ(29)+%11δ(53)
#65	525.54388	-
#66	498.10844	%11δ(46)+%12δ(50)
#67	476.92936	%12τ(68)

Table. S7. Assignment of normal mode of BiBz-2.

Mode	Frequency	Vibration assignment (>10%)
#1	3074.35	%11ν(1)+%11ν(4)+%24ν(5)+%11ν(12)+%11ν(13)+%24ν(14)
#2	3074.186	%11ν(1)+%11ν(4)+%25ν(5)+%11ν(12)+%11ν(13)+%24ν(14)
#3	3061.915	%21ν(1)+%21ν(4)+%21ν(12)+%21ν(13)
#4	3061.818	%21ν(1)+%21ν(4)+%21ν(12)+%21ν(13)
#5	3053.422	%22ν(5)+%22ν(14)
#6	3053.306	%22ν(5)+%22ν(14)
#7	3040.302	%21ν(2)+%22ν(3)+%21ν(10)+%21ν(11)
#8	3039.887	%21ν(2)+%22ν(3)+%21ν(10)+%22ν(11)
#9	3039.058	%17ν(2)+%16ν(3)+%17ν(10)+%16ν(11)

#10	3038.904	%17ν(2)+%16ν(3)+%17ν(10)+%16ν(11)
#11	2972.851	%25ν(6)+%25ν(7)+%25ν(8)+%25ν(9)
#12	2949.927	%25ν(6)+%25ν(7)+%25ν(8)+%25ν(9)
#13	2929.307	%25ν(6)+%25ν(7)+%25ν(8)+%25ν(9)
#14	2919.522	%25ν(6)+%25ν(7)+%25ν(8)+%25ν(9)
#15	1591.121	%15ν(16)+%15ν(19)
#16	1590.581	%15ν(16)+%15ν(19)
#17	1570.25	%15ν(18)+%13ν(22)+%12ν(23)+%10ν(24)
#18	1570.25	%13ν(18)+%16ν(22)+%10ν(23)+%12ν(24)
#19	1475.151	-
#20	1474.853	-
#21	1452.43	%45δ(36)+%45δ(38)
#22	1435.733	%42δ(36)+%42δ(38)
#23	1433.873	%13δ(34)+%13δ(43)
#24	1433.053	%13δ(34)+%13δ(43)
#25	1315.976	%17τ(59)+%20τ(60)+%11τ(61)+%20τ(62)
#26	1314.366	%10δ(31)+%10δ(32)+%10δ(39)+%10δ(40)
#27	1310.23	%13δ(31)+%13δ(32)+%13δ(39)+%13δ(40)
#28	1295.703	-
#29	1295.201	%10ν(17)+%10ν(20)+%10ν(23)+%10ν(24)
#30	1250.732	%14τ(59)+%20τ(60)+%20τ(62)
#31	1248.274	%25δ(37)+%11τ(60)+%11τ(62)
#32	1185.72	%18ν(25)+%17ν(26)
#33	1185.431	%18ν(25)+%18ν(26)
#34	1159.721	%10δ(31)+%10δ(32)+%10δ(39)+%10δ(40)
#35	1159.374	%10δ(31)+%10δ(32)+%10δ(39)+%10δ(40)
#36	1140.354	%20δ(34)+%20δ(43)
#37	1139.699	%10δ(33)+%19δ(34)+%10δ(41)+%10δ(42)+%19δ(43)
#38	1122.443	%25δ(35)+%22δ(37)
#39	1072.489	-
#40	1051.435	-
#41	1015.497	%11ν(15)+%11ν(18)+%11ν(21)+%11ν(22)
#42	1015.073	%11ν(15)+%11ν(18)+%11ν(21)+%11ν(22)
#43	985.555	%14δ(28)+%10δ(44)+%14δ(45)+%10δ(49)
#44	985.0441	%14δ(28)+%10δ(44)+%14δ(45)+%10δ(49)
#45	982.7016	%60ν(27)
#46	964.2892	%22δ(35)+%10δ(37)+%13τ(61)
#47	963.5469	%11τ(54)+%15τ(58)+%15τ(67)
#48	962.4576	%11τ(54)+%16τ(58)+%16τ(67)
#49	941.105	%10τ(54)+%13τ(57)+%13τ(65)+%13τ(66)
#50	940.9508	%10τ(54)+%13τ(57)+%13τ(65)+%13τ(66)
#51	890.8324	%13τ(55)+%13τ(56)+%17τ(58)+%13τ(63)+%13τ(64)+%17τ(67)
#52	890.36	%13τ(67)+%12ν(27)+%10τ(56)+%13τ(58)+%10τ(63)+%10τ(64)
#53	833.3394	%10δ(44)+%10δ(49)

#54	824.1525	%12τ(54)+%13τ(55)+%14τ(56)+%12τ(57)+%13τ(63)+%13τ(64)
#55	824.1332	%12τ(65)+%13τ(55)+%13τ(56)+%12τ(66)+%14τ(63)+%14τ(64)
#56	780.493	%12ν(25)+%12ν(26)+%20δ(44)+%20δ(49)
#57	747.5627	%10δ(37)+%25τ(59)+%12τ(60)+%21τ(61)+%12τ(62)
#58	745.8082	%13τ(71)+%15τ(75)
#59	729.9312	%10τ(71)+%14τ(75)
#60	689.6938	%10τ(58)+%10τ(67)+%10τ(71)+%16τ(75)
#61	689.5588	%10τ(58)+%10τ(67)+%10τ(71)+%15τ(75)
#62	616.5166	%12δ(28)+%11δ(45)+%10δ(46)+%18δ(47)+%18δ(48)+%10δ(51)
#63	615.3501	%12δ(28)+%12δ(45)+%10δ(46)+%18δ(47)+%18δ(48)+%10δ(51)
#64	605.1028	%11δ(44)+%11δ(49)
#65	517.7355	-
#66	515.2387	-
#67	465.2746	%18τ(68)+%14γ(78)

4.3. Comparison of theoretical and experimental spectra

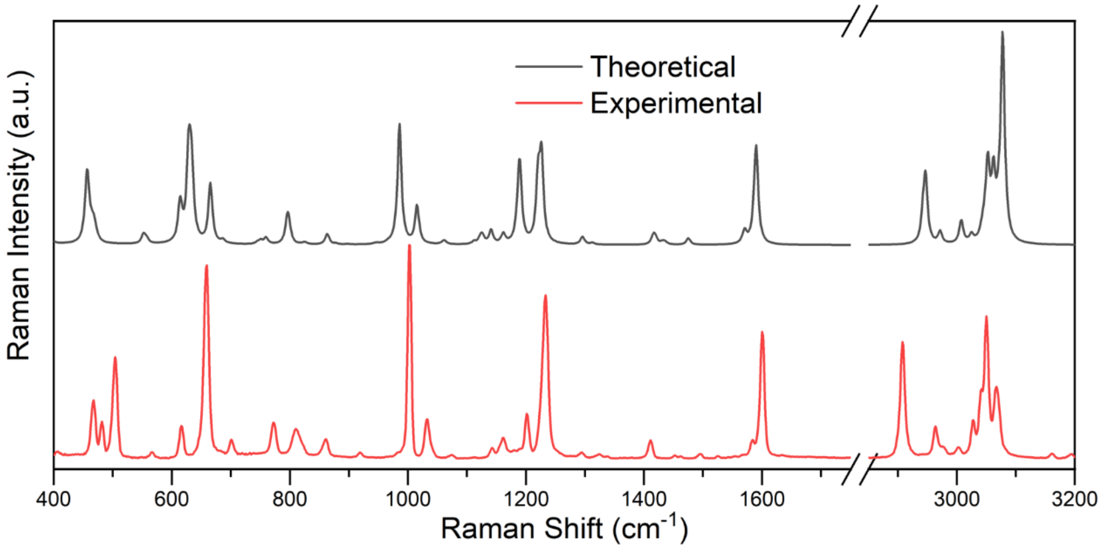

Fig. S4. Experimental measured and theoretically calculated Raman spectra of DBDS.

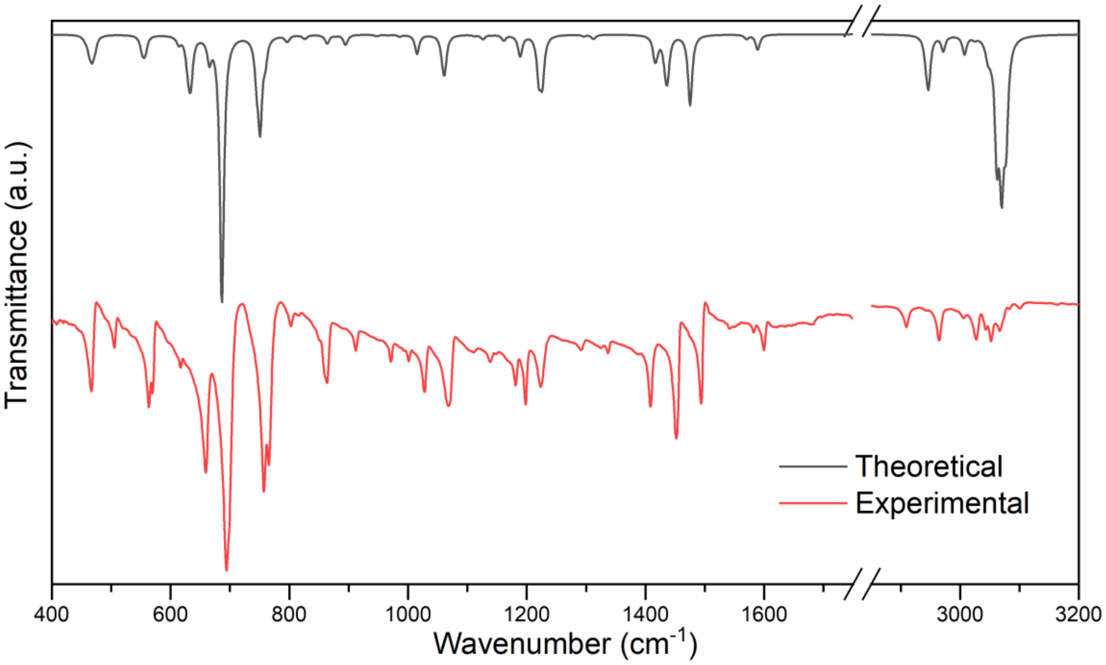

Fig. S5. Experimental measured and theoretically calculated IR spectra of DBDS.

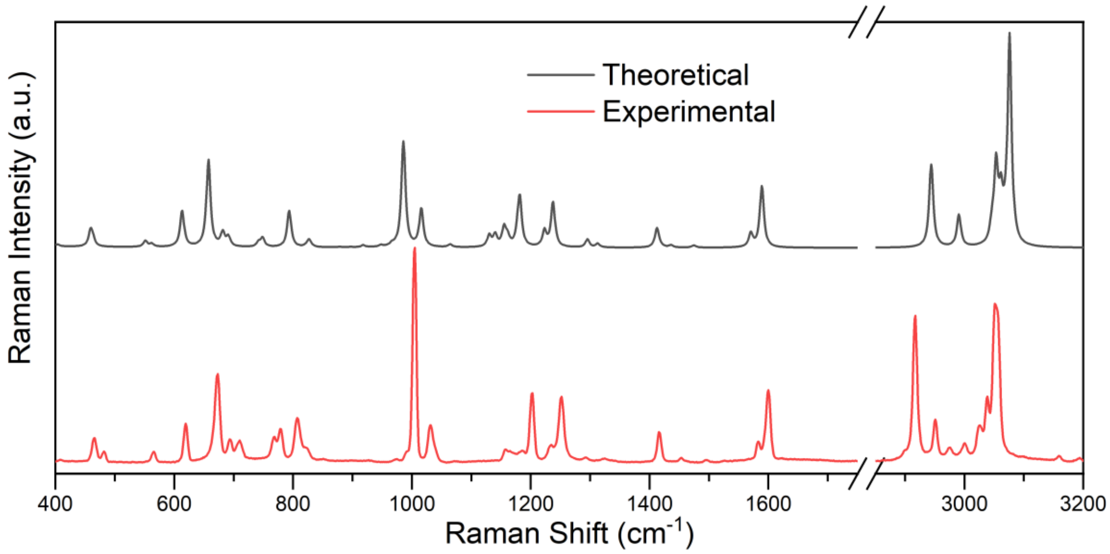

Fig. S6. Experimental measured and theoretically calculated Raman spectra of DBS.

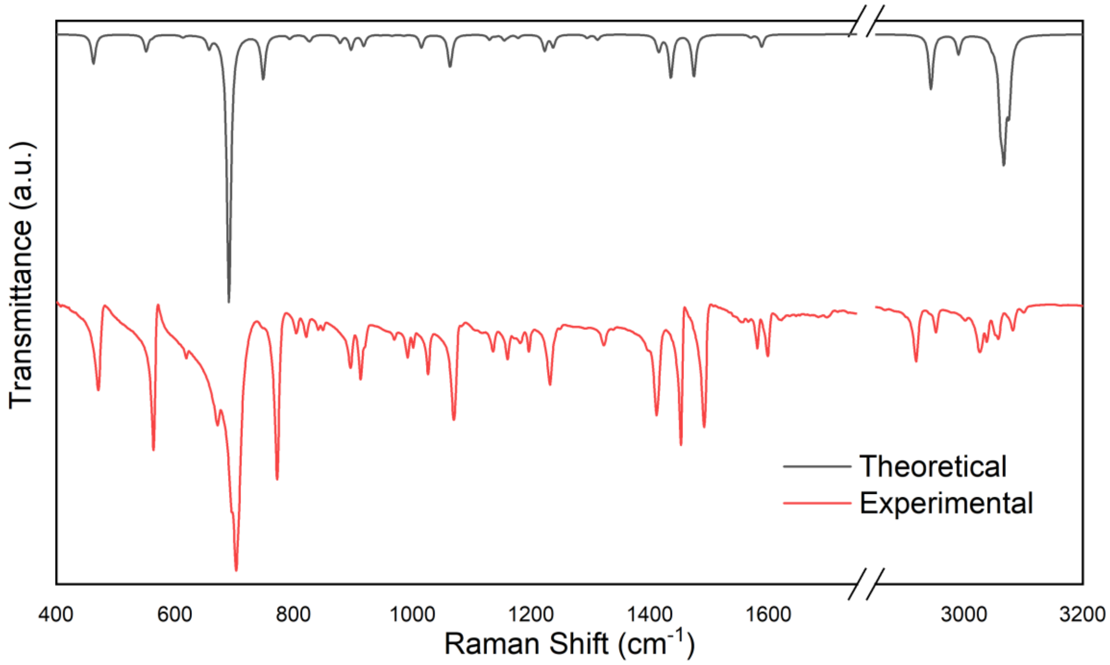

Fig. S7. Experimental measured and theoretically calculated IR spectra of DBS.

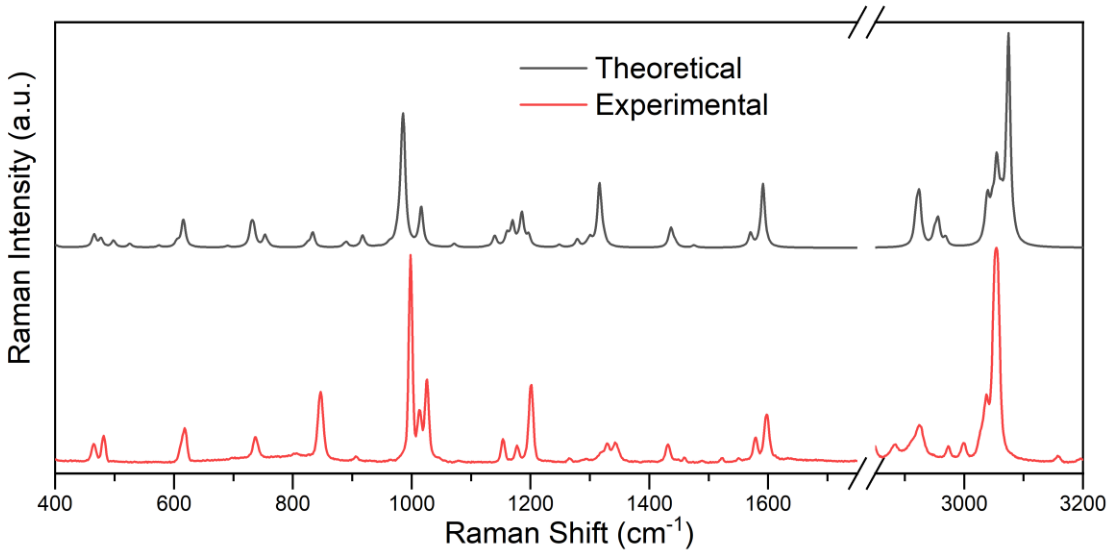

Fig. S8. Experimental measured and theoretically calculated Raman spectra of BiBz

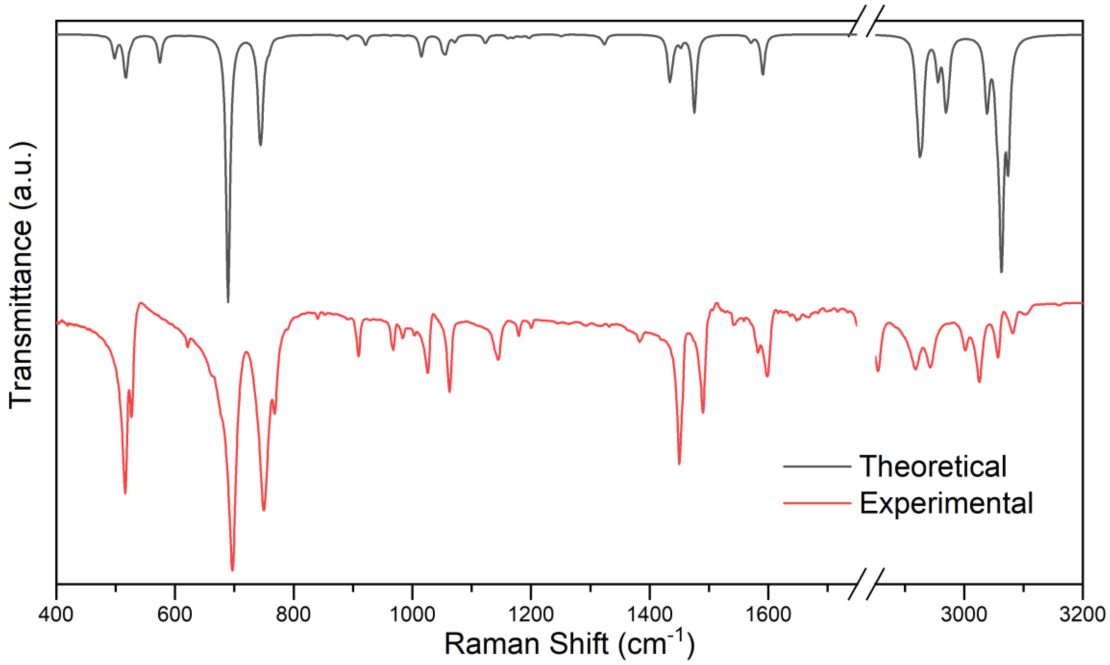

Fig. S9. Experimental measured and theoretically calculated IR spectra of BiBz
